# High mannose-specific lectin Msl mediates key interactions of the vaginal *Lactobacillus plantarum* isolate CMPG5300

**DOI:** 10.1038/srep37339

**Published:** 2016-11-17

**Authors:** Shweta Malik, Mariya I. Petrova, Nicole C. E. Imholz, Tine L. A. Verhoeven, Sam Noppen, Els J. M. Van Damme, Sandra Liekens, Jan Balzarini, Dominique Schols, Jos Vanderleyden, Sarah Lebeer

**Affiliations:** 1 KU Leuven, Centre of Microbial and Plant Genetics, Leuven, Belgium; 2 University of Antwerp, Department of Bioscience Engineering, Research Group Environmental Ecology and Applied Microbiology, Antwerp, Belgium; 3 KU Leuven, Department of Microbiology and Immunology, Rega Institute for Medical Research, Laboratory of Virology and Chemotherapy, Leuven, Belgium; 4 Ghent University, Department of Molecular Biotechnology, Ghent, Belgium

## Abstract

To characterize the interaction potential of the human vaginal isolate *Lactobacillus plantarum* CMPG5300, its genome was mined for genes encoding lectin-like proteins. *cmpg5300.05_29* was identified as the gene encoding a putative mannose-binding lectin. Phenotypic analysis of a gene knock-out mutant of *cmpg5300.05_29* showed that expression of this gene is important for auto-aggregation, adhesion to the vaginal epithelial cells, biofilm formation and binding to mannosylated glycans. Purification of the predicted lectin domain of Cmpg5300.05_29 and characterization of its sugar binding capacity confirmed the specificity of the lectin for high- mannose glycans. Therefore, we renamed Cmpg5300.05_29 as a mannose-specific lectin (Msl). The purified lectin domain of Msl could efficiently bind to HIV-1 glycoprotein gp120 and *Candida albicans,* and showed an inhibitory activity against biofilm formation of uropathogenic *Escherichia coli*, *Staphylococcus aureus* and *Salmonella* Typhimurium. Thus, using a combination of molecular lectin characterization and functional assays, we could show that lectin-sugar interactions play a key role in host and pathogen interactions of a prototype isolate of the vaginal *Lactobacillus* microbiota.

Given the key importance of the urogenital microbiota for human reproduction, increasing efforts are put in place to characterize this microbiome in healthy and pathogenic or unwanted conditions such as bacterial vaginosis (BV) and preterm labor^1^. Compared to microbial communities at other human body sites, the vagina provides an interesting case since dominance by specific bacterial taxa - namely *Lactobacillus* species- is clearly linked to a healthy state^2^. These lactobacilli appear to promote the protection of the urogenital tract from viral pathogen invasion such as Human Immunodeficiency Virus (HIV)^2^,^3^ and Herpes Simplex virus-2 (HSV-2)^4^, bacterial and yeast pathogens such as *Escherichia coli*^5^ and *Staphylococcus aureus*^6^ and *Candida albicans*^7^, respectively. However, the molecular mechanisms behind the health-promoting effects of vaginal lactobacilli are in general not well known. One of the postulated health benefits of lactobacilli is based on the direct inhibition of pathogens by the production of active compounds such as lactic acid and bacteriocins (Petrova *et al.*^2^). Another mechanism by which lactobacilli are known to prevent infections is by preventing adhesion, invasion and biofilm formation of the pathogens. For example, vaginal lactobacilli have been suggested to be able to prevent and/or treat *E. coli-*associated urinary tract infections (UTI) and aerobic vaginitis^8^,^9^, but the molecules involved are not well studied. Cell surface molecules of lactobacilli could play a role in pathogen exclusion by (1) competitive binding to the same receptors as pathogens on the host surface, thereby blocking pathogen adhesion or (2) by binding to ligands on the pathogenic surfaces, thereby blocking virulence mechanisms such as invasion and/or promoting enhanced exposure to the secreted antimicrobials of lactobacilli^2^.

Lectins are specific sugar-binding proteins or carbohydrate-binding agents (CBAs). Because of the key role of sugar recognition in many physiological functions, they can be postulated to mediate crucial functions of the human microbiota, including colonization of the host and interaction with pathogens^10^. Yet, they are underexplored in functional microbiota studies. In this study, we investigated the lectin-sugar interaction potential of a vaginal microbiome isolate, *Lactobacillus plantarum* CMPG5300, for which we recently determined the genome sequence^11^. Genome mining of *L. plantarum* CMPG5300 resulted in the identification of *cmpg5300.05_29* as the gene encoding a putative mannose-binding lectin.

## Results

### Identification of the putative lectin-like encoding *cmpg5300.05_29* gene on a ~28 kb plasmid

When we mined the genome sequence of *L. plantarum* CMPG5300 for putative lectin-encoding genes (Malik *et al.*^11^), the sortase-dependent, putative mannose-specific adhesin-encoding gene *cmpg5300.05_29* of CMPG5300 was found to be located at the start and the end of a contig (contig 54) indicating the contig was circular, hence a plasmid. The gene *cmpg5300.05_29* shows 72% identity to the mannose specific adhesin (*msa*) gene *lp_1229* of *Lactobacillus plantarum* WCFS1^12^ at DNA level, while the encoded proteins show 61% similarity (5e-55). However, Cmpg5300.05_29 is 193 amino acids longer than that of *L. plantarum* WCFS1 (Lp_1229). The protein consists of a ConA-like domain, classified as a Legume-L type lectin followed by Type I MUB domain and four copies of Type II MUB domains.

**Figure 1:**
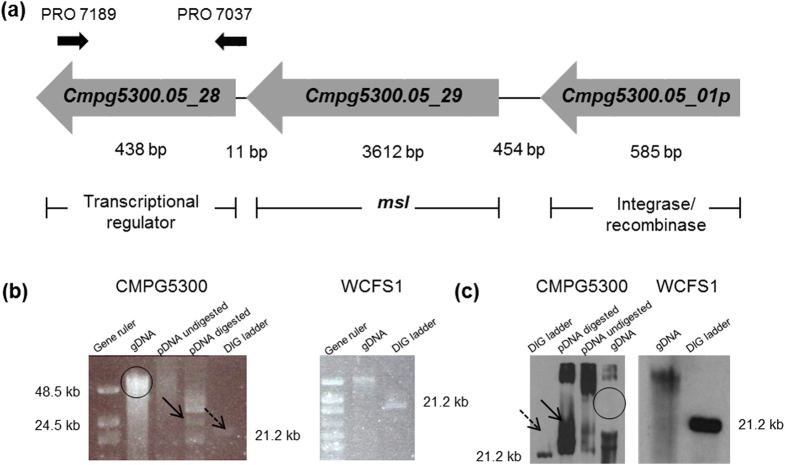
Plasmid location of *cmpg5300.05_29*. (**a**) Schematic illustration of the genetic organization of *cmpg5300.05_29* on the putative plasmid contig 54. The primer pair PRO 7189 and PRO 7037 (black arrows) was used for constructing the probe for Southern hybridization. (**b**) Southern blot analysis. DNA gel profile for CMPG5300- Lane Gene ruler: High range ladder; Lane gDNA: Total genomic DNA preparation (band encircled in black); Lane pDNA undigested: Total plasmid DNA preparation-undigested (500 ng/ml); Lane pDNA digested: Total plasmid DNA digested with restriction enzymes (500 ng/ml); Lane DIG ladder: DIG ladder. DNA gel profile for WCFS1- Lane Gene ruler: High range ladder; Lane gDNA: Total genomic DNA; Lane DIG ladder: DIG ladder. (**c**) Southern blot of CMPG5300- Lane DIG ladder: DIG ladder; Lane pDNA digested: Total plasmid DNA digested (500 ng/ml); Lane pDNA undigested: Total plasmid DNA-undigested (500 ng/ml); Lane gDNA: Total genomic DNA. Southern blot of WCFS1- Lane gDNA: Total genomic DNA; Lane DIG ladder: DIG ladder. Southern hybridization of a 487-bp *cmpg5300.05_28*-specific labeled fragment gave a binding signal (at a size slightly higher than 21.2 kbp) marked in black solid arrow (in both DNA gel and Southern blot pictures) with the plasmid DNA preparations indicating the plasmid location of *cmpg5300.05_29.* No signal was obtained at the position of the total DNA (encircled). On the other hand, the 772-bp labeled fragment of *L. plantarum* WCFS1 (used as control) yielded a signal corresponding to band of the chromosomal DNA preparation.

Validation of the plasmid location was done experimentally, with PCR and Southern hybridization with various primers mentioned in Table S1 and probes depicted in Fig. 1a. Since the *cmpg5300.05_29* gene itself contains a lot of repetitions, we focused on detecting the regulator encoding gene *cmpg5300.05_28* located upstream from this sequence. Southern hybridization indeed confirmed the presence of the genes *cmpg5300.05_28* and *cmpg5300.05_29* mainly in the plasmid DNA samples (Fig. 1b and c). The size of the plasmid hybridizing with *cmpg5300.05_28* also corresponds to the size of contig 54 on which *cmpg5300.05_29* is located, which is ~28 kb. This plasmid location is unlike the chromosomally-located homologous *msa* genes in other *L. plantarum* strains^13^, as confirmed here for *L. plantarum* strain WCFS1 (Fig. 1b and c).

### Mutant analysis shows a key role for Cmpg5300.05_29 in auto-aggregation and adhesion

**Figure 2:**
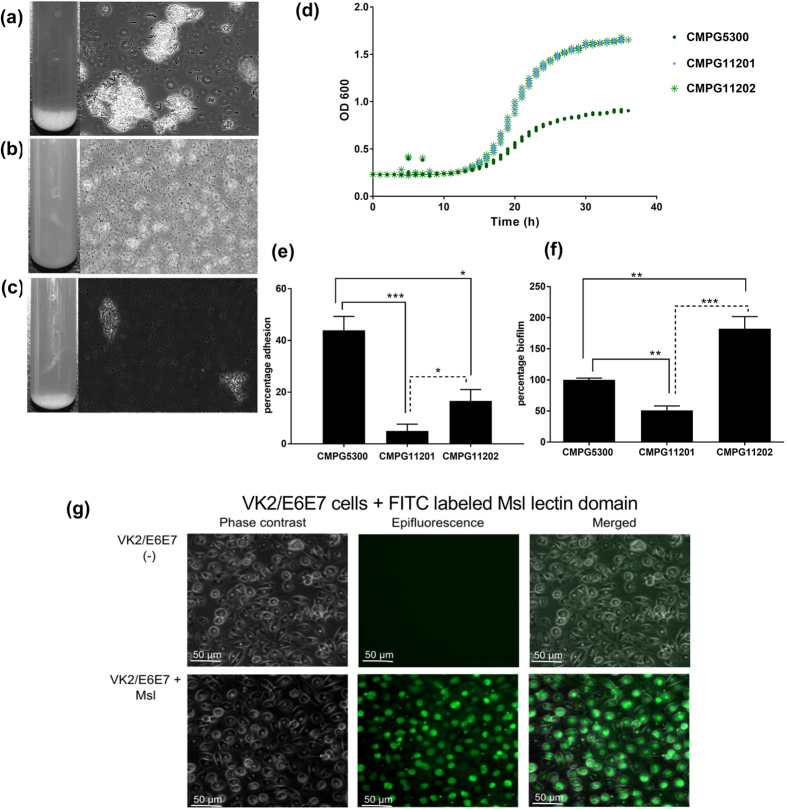
Phenotypic analysis of the mutant strain CMPG11201. (**a**) Overnight cultures of *L. plantarum* CMPG5300 (**b**) its mutant CMPG11201, and (**c**) complemented strain CMPG11202 in MRS medium. Phase-contrast microscopic images show extensive cell clumps in the case of *L. plantarum* CMPG5300 (**a**) and some clumps in the complemented strain CMPG11202 (**c**), in contrast to the small-sized clusters and mostly single cells that are seen with the mutant CMPG11201 (**b**). (**d**) Growth capacity of *L. plantarum* CMPG5300, CMPG11201 and CMPG11202 mutants in MRS medium. (**e**) Adhesion capacities of *L. plantarum* CMPG5300 and CMPG11201 mutant to VK2/E6E7 vaginal epithelial cells. (**f**) Biofilm formation capacities of *L. plantarum* CMPG5300 and mutant CMPG11201. The results are expressed relative to the biofilm thickness of *L. plantarum* CMPG5300 wild-type (positive control), which was set at 100%. The error bars represent standard deviations of 8 biological repeats. The dataset comparisons (mutant pairwise to wild-type or complemented strain pairwise mutant strain) are considered significant (p < 0.05 indicated with one asterisks, p < 0.01 indicated with two asterisks and p < 0.001 indicated with three asterisks in the figure). (**g**) Binding of the FITC-labelled lectin domain of Msl to VK2/E6E7.

Subsequently, the *cmpg5300.05_29* knock-out mutant designated CMPG11201 was constructed by double homologous recombination. This mutant strain CMPG11201 did not display extensive cell clumping, as observed for the wild-type CMPG5300 (Fig. 2a and b). In contrast, the complemented strain CMPG11202 exhibited cell clumps, nonetheless smaller than the wild-type *L. plantarum* CMPG5300, suggesting that the phenotype was not fully restored (Fig. 2c). Of note, the mutant showed the same growth characteristics as wild-type strain *L. plantarum* CMPG5300 when grown in MRS (Fig. 2d). *L. plantarum* CMPG5300 and mutant CMPG11201 were then analyzed for adhesion to the vaginal epithelial cell line VK2/E6E7 (Fig. 2e) and biofilm formation (Fig. 2f) on a polystyrene surface. A significant decrease of ~80 percent was observed for adhesion and ~50 percent for biofilm formation in case of the mutant strain CMPG11201 compared to the wild-type. The restoration of the phenotype of the complemented strain CMPG11202 substantiates the role of this lectin in the adhesive and biofilm-forming phenotype of the strain (Fig. 2e and f). Of note, the adhesive capacity of the complemented strain was not fully restored to the wild-type level like the biofilm-forming phenotype although it was significantly higher than that of the mutant. To validate the role of Msl in the adhesion capacity of the *L. plantarum* CMPG5300, the FITC- labelled lectin domain of Msl was examined for binding to VK2/E6E7 using fluorescence microscopy. The results show clear binding of the lectin domain of Msl to VK2/E6E7 epithelial cells (Fig. 2g).

### *Saccharomyces cerevisiae* and *Candida albicans* agglutination assay shows mannan-binding capacity of Cmpg5300.05_29

**Figure 3:**
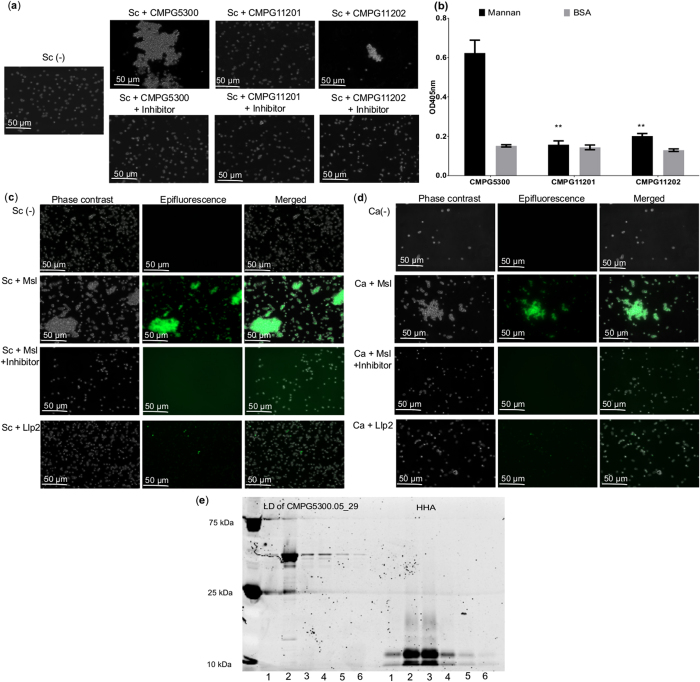
Agglutination assay of *L. plantarum* CMPG5300, its mutant derivatives and the lectin domain of Cmpg5300.05_29 with *S. cerevisiae* and *C. albicans*. (**a**) Phase contrast images of the agglutination assay of *S. cerevisiae* BY4741 (Sc) incubated with *L. plantarum* CMPG5300, CMPG11201 mutant and CMPG11202 mutant alone and in the presence of methyl-α-D-mannopyranoside inhibitor. For each strain and condition tested, the experiment was performed at least three times. The images shown are representative of at least five imaging fields. (**b**) Binding of biotin-labeled cells of the wild-type strain *L. plantarum* CMPG5300, mutant CMPG11201 and complemented strain CMPG11202 to yeast mannan. The OD at 405 nm reflects the binding efficiency of the bacterial strains. BSA was used as a negative control. The dataset comparisons (mutant pairwise to wild-type) are considered significant (p < 0.01 indicated with two asterisks in the figure). The dataset comparison (complemented mutant pairwise to the mutant strain) show no significant differences. (**c**) Fluorescent images of the agglutination assay of *S. cerevisiae* BY4741 (Sc) and (**d**) *C. albicans* SC5314 (Ca) in the presence of the FITC labeled lectin domain of Cmpg5300.05_29, FITC labeled lectin domain of Cmpg5300.05_29 in the presence of methyl-α-D-mannopyranoside and lectin-like protein 2 (Llp2) from *L. rhamnosus* GG used as control. For each strain and condition tested, the experiment was performed at least three times. The images shown are representative of at least five imaging fields. (**e**) Proteins that bound to uncoated sepharose beads (lane 1, used as negative control), sepharose beads coated with various sugars: mannan (lane 2), mannose (lane 3), glucose (lane 4), fucose (lane 5), GlcNAc (lane 6) as separated by SDS-PAGE. The first six lanes (LD of CMPG5300.05_29) represent the lectin domain of CMPG5300.05_29 and the next six lanes represent HHA (used as control).

To obtain a first indication of the sugar specificity of Cmpg5300.05_29, a *S. cerevisiae* BY4741 agglutination assay was performed, which is a standard assay to explore mannose-binding activity of bacterial lectins^14^. *L. plantarum* CMPG5300 showed a clear capacity to agglutinate *S. cerevisiae*, which was independent of the presence of calcium and magnesium ions (data not shown) and this was reduced significantly by addition of methyl-α-D-mannopyranoside (Fig. 3a). In contrast, mutant CMPG11201 showed a significant decrease in agglutination (Fig. 3a), while the complemented strain CMPG11202 was also able to agglutinate *S. cerevisiae* to some extent (Fig. 3a). We could corroborate these findings using ELISA where we obtained a significant binding of the wild-type strain CMPG5300 to mannan but a clear loss of binding for the *cmpg5300.05_29* mutant (Fig. 3b). To exclude that other cell surface ligands could still interfere with the results in the assay with the mutant strain, we subsequently expressed and purified the lectin domain of Cmpg5300.05_29. Only the predicted lectin domain was expressed in *E. coli*, as we were unable to express the large-sized protein (encoded by a ~3.6 kb long gene). The purified lectin domain could also agglutinate *S. cerevisiae* (Fig. 3c) and the related yeast pathogen *C. albicans* (Fig. 3d), as shown after FITC labeling, providing an indication for the potential interaction of the lectin with pathogens. This agglutination was significantly reduced in the presence of methyl-α-D-mannopyranoside (Fig. 3c and d). The L-type lectin domain of lectin-like protein 2 (Llp2) isolated from *Lactobacillus rhamnosus* GG used as a control did not agglutinate *S. cerevisiae* and *C. albicans* (Fig. 3c and d).

### Cmpg5300.05_29 shows specificity for high-mannose N-glycans

To determine the sugar specificity of Cmpg5300.05_29 in a more precise manner, pull-down sugar-binding assays were performed using Sepharose beads coated with various sugar substrates. The well documented mannose-specific *Hippeastrum* hybrid (amaryllis) lectin (HHA)^15^ was used as a positive control. These experiments showed that the lectin domain of Cmpg5300.05_29 binds more strongly to beads coated with mannan compared to uncoated beads and beads coated with mannose, glucose, fucose or GlcNAc (Fig. 3e).

**Figure 4:**
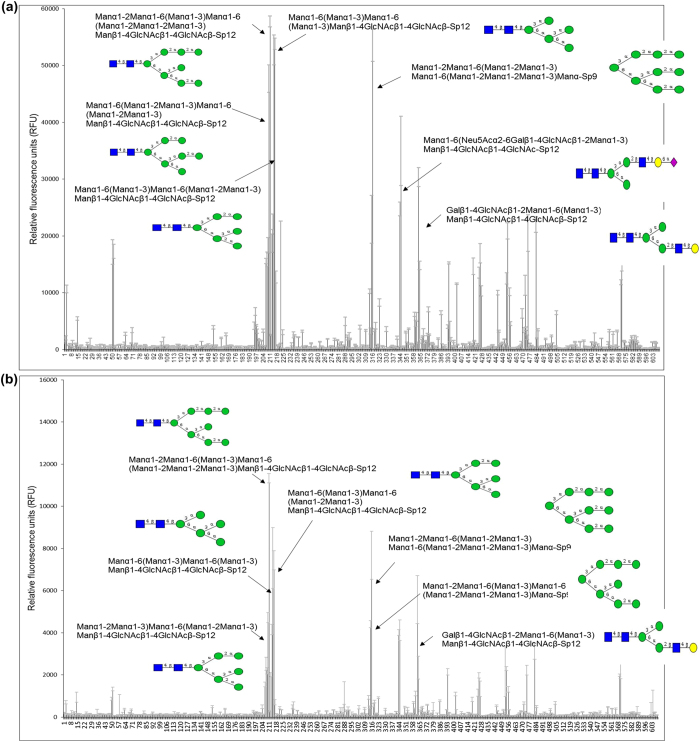
Glycan array analysis. (**a**) Binding capacity of 200 μg/ml of the lectin domain of Msl to high-mannose glycans. (**b**) Binding capacity of 20 μg/ml of the lectin domain of Msl to high-mannose glycans as depicted with black arrows in the figure (the structure of the glycans printed on the array can be find on the webpage of the CFG, www.functionalglycomics.org) and in Table S2.

The glycan array analysis revealed that the lectin domain binds to especially high mannose N-type glycans (Fig. 4a and b, Table S2). These include Manα1-2Manα1-6(Manα1-3)Manα1-6(Manα1-2Manα1-2Manα1-3)Manβ1-4GlcNAcβ1-4GlcNAcβ-Sp12; Manα1-6(Manα1-3)Manα1-6(Manα1-3)Manβ1-4GlcNAcβ1-4GlcNAcβ-Sp12; Manα1-2Manα1-6(Manα1-2Manα1-3)Manα1-6(Manα1-2Manα1-2Manα1-3)Manα-Sp9 and Manα1-6(Manα1-3)Manα1-6(Manα1-2Manα1-3)Manβ1-4GlcNAcβ1-4GlcNAcβ-Sp12.

### Interaction between Cmpg5300.05_29 and HIV-1 glycoproteins

**Figure 5:**
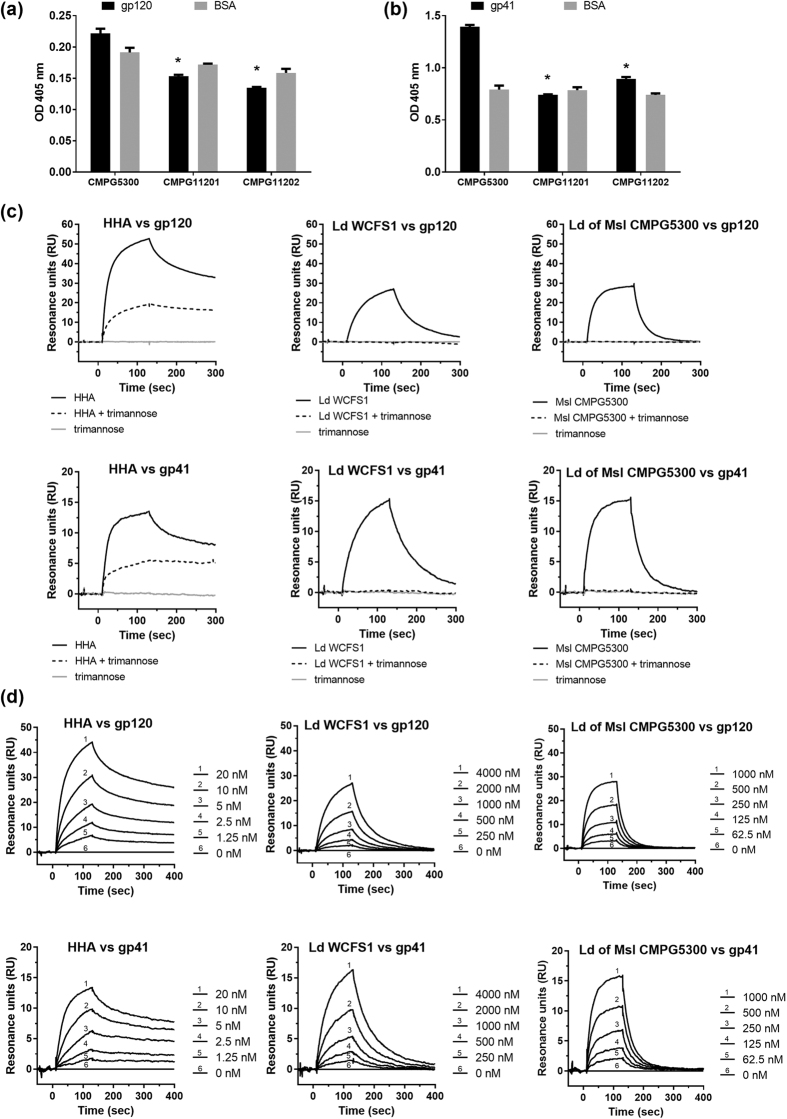
HIV-1 gp120 and gp41 binding of bacterial cells and the purified lectin domain of Cmpg5300.05_29. (**a**) Binding of biotin-labeled cells of the wild-type strain *L. plantarum* CMPG5300, mutant CMPG11201 and complemented strain CMPG11202 to HIV-1 gp120. The OD at 405 nm reflects the binding efficiency of the bacterial strains. BSA was used as a negative control. (**b**) Binding of biotin-labeled cells of the wild-type strain *L. plantarum* CMPG5300, mutant CMPG11201 and complemented strain CMPG11202 to HIV-1 gp41. The OD at 405 nm reflects the binding efficiency of the bacterial strains. BSA was used as a negative control. (**c**) Binding of the lectin domain of CMPG5300 (Ld CMPG5300) to HIV-1 gp120 (solid lines) and HIV-1 gp41 (solid lines) in terms of Resonance units as analyzed via SPR. The purified lectin domain of Msa of *L. plantarum* WCFS1 (Ld WCFS1) was used as control (solid lines) and HHA was used as positive control (solid lines). Upon addition of competitor trimannose Manα(1-3)Manα(1-6)Man to the lectin domains, binding to HIV-1 gp120 was abolished (dotted lines). The concentrations of the lectins used are 20 nM, 4 μM and 1 μM for HHA (left), lectin domain of Msa of *L. plantarum* WCFS1 (centre) and lectin domain of CMPG5300.05_29 of CMPG5300 (right), respectively. A concentration of 1 mM was used for trimannose. (**d**) Kinetic analysis for the interaction of the lectin domain of CMPG5300.05_29 with HIV-1 gp120 and gp41 using the lectin domain of Msa and HHA as control. Serial two-fold analyte dilutions (covering a concentration range from 0 to 20 nM for HHA, 0 to 4000 nm for Ld WCFS1 and from 0 to 1000 nM for Ld CMPG5300) were injected over the surface of the immobilized gp120 and gp41. A 1:1 binding model was applied to determine the kinetic parameters. The biosensor chip density was 115 RUs for gp120 and 45 RUs for gp41. The dataset comparisons (mutant pairwise to wild-type) are considered significant (p < 0.05 indicated with one asterisks). The dataset comparison (complemented mutant pairwise to the mutant strain) show no significant differences.

Since the HIV-1 glycoproteins are enriched in high-mannose N-type glycans^16^, we determined the interaction of *L. plantarum* CMPG5300 and the lectin domain of Cmpg5300.05_29 with HIV. First, the capacity of CMPG5300 and its mutant derivatives to bind to immobilized HIV-1 gp120 and gp41 was analyzed using an ELISA-based approach. The wild-type strain *L. plantarum* CMPG5300 showed a significant and specific binding to both gp120 and gp41 (Fig. 5a and b). Whereas, the mutant CMPG11201 did not show any binding, suggesting that Cmpg5300.05_29 imparts the gp120 and gp41 binding phenotype to *L. plantarum* CMPG5300. However, the binding to these sugars could not be restored in the complemented strain CMPG11202. Subsequently, we confirmed binding of the lectin domain to HIV-1 gp120 with Surface Plasmon Resonance (SPR) (Fig. 5c). This was reversed by 1 mM manα(1-3)manα(1-6)man (dotted lines, Fig. 5b), indicating that this trimannose is a competitive inhibitor for binding of the lectin domain to the HIV glycoproteins, and that the lectin domain shows affinity for the α(1-3)/α(1-6) configuration of mannose. The kinetic results show affinities (*K*_*D*_) in the lower micromolar range (comparable for both gp120 and gp41) (Fig. 5d). Comparison of the binding affinities of the lectin domains of *L. plantarum* CMPG5300 (1.85E-06 M) and *L. plantarum* WCFS1 (6.26E-06 M) (Table 1) indicates that the lectin domain of *L. plantarum* CMPG5300 shows a higher affinity to both HIV-1 gp120 and gp41, than that of *L. plantarum* WCFS1, which is not a vaginal isolate. However, HHA (used as a positive control) shows a 1,000-fold higher affinity (or 1,000-fold lower K_D_) (1.32E-09 M) for the viral envelope glycoproteins.

Given the binding capacity of the lectin domain to HIV-1 gp120 (and gp41) observed in the SPR experiments, it was of interest to determine its capacity to inhibit HIV-1 infection in cell culture. However, addition of the purified lectin domain even up to a concentration of 200 μg/ml was not sufficient to measurably inhibit HIV-1 infection in human CD4+ T-lymphocytic C8166 cell and Raji DCSIGN cultures under our experimental conditions where HHA and GRFT were clearly inhibitory at concentrations less than 1 μg/ml (data not shown).

### Anti-biofilm activity of the lectin domain of Cmpg5300.05_29 against common urogenital bacterial pathogens

**Figure 6:**
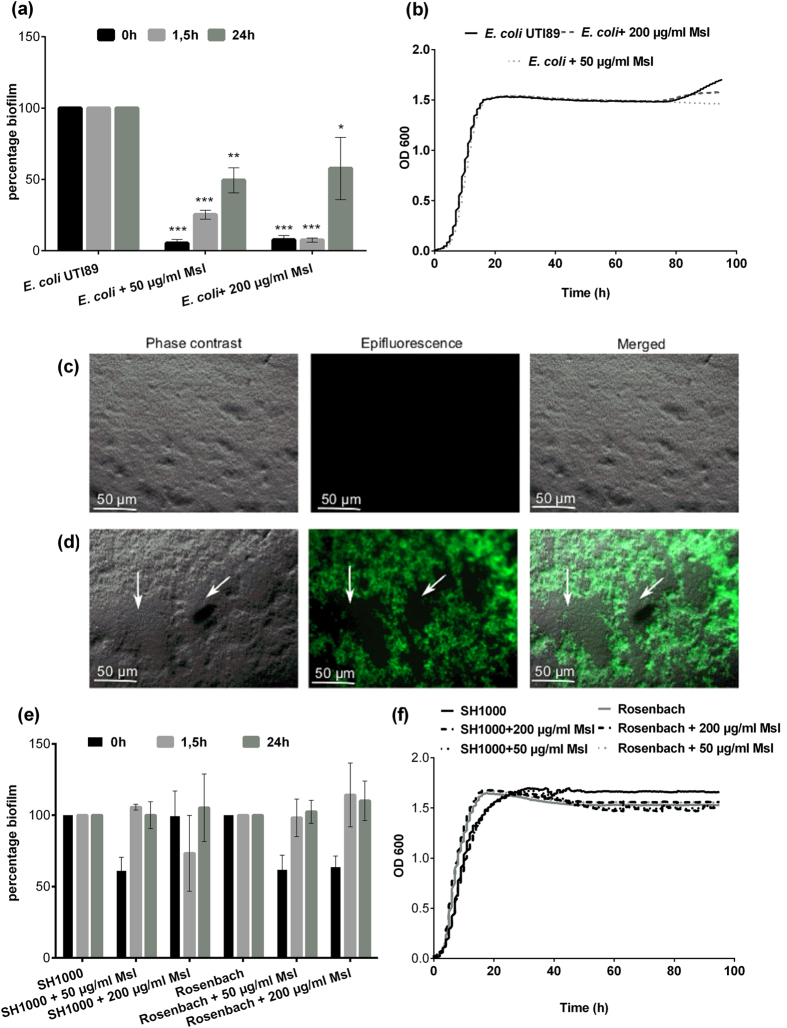
Activity of the lectin domain of Cmpg5300.05_29 against *E. coli* UTI89, *S. aureus* SH1000 and Rosenbach. (**a**) Anti-biofilm activity of the lectin domain of Cmpg5300.05_29 against *E. coli* UTI89 expressed as percentage inhibition of biofilm formation. The purified lectin domain of Cmpg5300.05_29 was added after 0, 1.5 and 24 h to the biofilms at a concentration of 50 μg/ml and 200 μg/ml. (**b**) Growth of *E. coli* UTI89 in TSB in the presence of lectin domain of Cmpg5300.05_29 added at 50 μg/ml and 200 μg/ml. (**c**) Microscopic images of *E. coli* UTI89 biofilms grown only in 1/20 TSB and (**d**) with 50 μg/ml of FITC-labeled lectin domain of Cmpg5300.05_29. Holes in the biofilms are indicated with arrows. (**e**) Anti-biofilm activity of the lectin domain of Cmpg5300.05_29 against *S. aureus* SH1000 and Rosenbach. The purified lectin domain of Cmpg5300.05_29 was added after 0, 1.5 and 24 h to the biofilms at concentration of 50 μg/ml and 200 μg/ml. (**f**) Growth of *S. aureus* SH1000 and Rosenbach in TSB in presence of lectin domain of Cmpg5300.05_29 added at 50 μg/ml and 200 μg/ml. The dataset comparisons (biofilm formation with addition of lectin to biofilm without adding lectins) are considered significant (p < 0.05 indicated with one asterisks, p < 0.01 indicated with two asterisks and p < 0.001 indicated with three asterisks in the figure).

We subsequently focused on the potential of Cmpg5300.05_29 for preventing bacterial infections by investigating its role in inhibiting bacterial biofilms. When the purified lectin domain of Cmpg5300.05_29 was added to biofilm cultures of uropathogenic *Escherichia coli* UTI89 at the onset of biofilm development (t = 0 h), it significantly inhibited biofilm formation of the pathogen by 80 to 90 percent at 50 μg/ml and 200 μg/ml, respectively (Fig. 6a). When added after 1.5 h or 24 h of biofilm development, it could reduce biofilm formation up to 60 percent compared to the control, indicating that it could also interfere with the later steps of biofilm formation (Fig. 6a). To corroborate the specific activity of Cmpg5300.05_29, ConA and HHA, well-known mannose-specific plant lectins, were also investigated for their activity against *E. coli* UTI89 biofilms but no inhibition was observed (Fig. 1Sa). In addition, since *E. coli* UTI89 biofilm formation is based on mannose-specific binding of FimH fimbria, we used D-mannose as a competitive inhibitor in the assay and again, no inhibition of biofilm formation was observed (Fig. 1Sa). Of note, we could not find any effect of the lectin on the planktonic growth of *E. coli* UTI89 under the same growth conditions as during the biofilm formation (Fig. 6b), indicating that the inhibition is biofilm-dependent. To explore how the lectin domain structurally interferes with *E. coli* UTI89 biofilm formation, microscopic analyses of the biofilms incubated with the lectin were performed. The results show that addition of FITC-labeled lectin domain of Cmpg5300.05_29 at the onset of the biofilm at concentration 50 μg/ml led to the formation of large holes in the biofilm (Fig. 6d), as compared to the negative control where dense biofilms were seen (Fig. 6c). The lectin domain of Cmpg5300.05_29 was also dispersed within the biofilm (Fig. 6d).

The lectin domain of Cmgp5300.05_29 subsequently showed the capacity to inhibit biofilm formation by *Staphylococcus aureus*, a pathogen causing the fatal toxic shock syndrome, with an average biofilm reduction up to 36 percent in the case of *S. aureus* strain SH1000 and 41 percent in the case of *S. aureus* Rosenbach when added at the onset of the biofilm (t = 0 h) (Fig. 6e). However, when it was added after 1.5 h or 24 h of biofilm development, there was no significant anti-biofilm activity, indicating that for these pathogens the inhibition of biofilm formation is merely at the initial stage of adhesion. Bioscreen results showed no effect of the lectin domain on the planktonic growth of either strain (Fig. 6f).

### Anti-biofilm activity of the lectin domain of Cmpg5300.05_29 against non-urogenital pathogens

**Figure 7:**
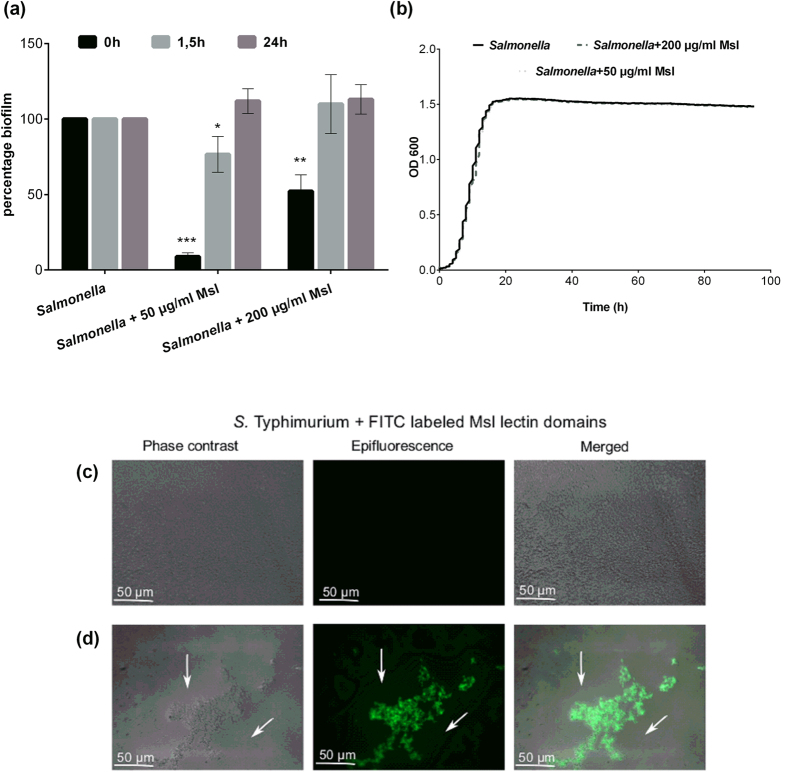
Activity of the lectin domain of Cmpg5300.05_29 against *S.* Typhimurium ATCC14028. (**a**) Anti-biofilm activity of the lectin domain of Cmpg5300.05_29. The purified lectin domain of Cmpg5300.05_29 was added after 0, 1.5 and 24 h to the biofilms at 50 μg/ml and 200 μg/ml. (**b**) Growth of *S.* Typhimurium ATCC14028 in presence of lectin domain of Cmpg5300.05_29 added at 50 μg/ml and 200 μg/ml. The dataset comparisons (biofilm formation with addition of lectin to biofilm without adding lectins) are considered significant (p < 0.05 indicated with one asterisks, p < 0.01 indicated with two asterisks and p < 0.001 indicated with three asterisks in the figure). (**c**) Biofilms of *S*. Typhimurium ATCC14028 grown in 1/20 TSB (negative control) and (**d**) with 50 μg/ml of FITC-labeled lectin domain of Cmpg5300.05_29. Holes in biofilms are indicated with arrows.

As for urogenital pathogens, various biofilm assays were also performed with the gastrointestinal pathogen *Salmonella* Typhimurium ATCC14028 and the opportunistic pathogen *Pseudomonas aeruginosa*. When the purified domain was added at a concentration of 50 and 200 μg/ml at the start of the static peg biofilm assay (t = 0 h), it led to a significant reduction in biofilm formation by an average of 78 percent (Fig. 7a) for *S*. Typhimurium, but not for *P. aeruginosa* PA14 (Fig. S1 b). No effect was seen when it was added in the later phases of biofilm development (Fig. 7a). Figure 7b demonstrates that even the planktonic growth was not impaired by the lectin domain of Cmpg5300.05_29. Similar to the assay of *E. coli* UTI89, the effect of ConA, HHA, and D-mannose (used as controls) against *Salmonella* Typhimurium ATCC14028 biofilms was also investigated where no inhibition was observed (Fig. 1Sa).

Furthermore, microscopic analyses of *S.* Typhimurium biofilms showed that the addition of FITC-labeled lectin domain of Cmpg5300.05_29 at the onset of the biofilm also resulted in the formation of large holes in the biofilm with the lectin domain of Cmpg5300.05_29 dispersed within the biofilm (Fig. 7c and d).

## Discussion

In this study, we genetically and biochemically identified and characterized a 127 kDa mannose-specific lectin Msl from a vaginal *Lactobacillus* isolate. The lectin was shown to mediate vaginal niche-related functions for *L. plantarum* CMPG5300 including adhesion to vaginal epithelial cells and auto-aggregation. In addition, the lectin domain showed the ability of structurally inhibiting biofilm formation by various pathogens, as well as a unique interacting capacity with yeast pathogens and viral envelope glycoproteins.

Combining mutant bacterial strains, purified proteins and lectin assays, we could clearly substantiate the high-mannose specificity of Msl. Inhibition of yeast agglutination with methyl-mannopyranoside, loss of mannan-binding capacity observed for the *msl* mutant, binding of the purified lectin domain to mannose-coated Sepharose beads, binding signal obtained with high-mannose N-glycans in the glycan array and competitive inhibition of HIV-1 gp120-binding by a trimer of mannose, all validate the specificity of our *L. plantarum* lectin to (oligo)mannoses. The fact that the lectin domain is even able to agglutinate yeast cells suggests that each lectin domain may bind to several ligands which can be also be predicted from its sequence to form dimmers/tetramers. This study reports on the detailed and unambiguous characterization of a lectin from a human vaginal *Lactobacillus* member with detailed glycan array studies showing clear sugar-binding activities. Glycan arrays have been implemented for the determination of the sugar specificity for only a limited number of putative bacterial lectins. For example, the soluble lectin from *P. aeruginosa* LecB (known as PA-IIL) has been shown to bind strongly to a large variety of fucosylated oligosaccharides, such as α-Fuc 1-2 Gal and β-Gal 1-4 α(Fuc1-3)GlcNAc^17^. The FimH adhesin located at the tip of type 1 pili of the uropathogenic *E. coli* (UPEC) play a role in attachment to urothelium by binding to mannosylated glycoreceptors^18^. Furthermore, to the best of our knowledge, only one other L-type bacterial lectin, known as SraP, has been characterized^19^. SraP isolated from *S. aureus* was shown to have a high specificity for N-acetylneuraminic acid, as determined by SPR analyses, but not by glycan array, and appears to play an important role in the adhesion of the strain to human respiratory epithelial cells.

Intriguingly, the lectin-encoding *msl* gene, as determined by Southern blot hybridization, was found to be encoded by one of the plasmids of *L. plantarum* CMPG5300. To our knowledge, plasmid-encoded adhesins have so far not often been reported in lactobacilli except for a study on the probiotic intestinal strain *L. paracasei* NFBC338 which encodes for a collagen-binding protein (CD00222) on one of its plasmids^20^. In contrast, plasmid-encoded adhesins and virulence factors that are well-characterized are found especially in enteropathogenic strains such as *Yersinia* and *Salmonella*^21^. The plasmid location of the *msl* gene could suggest that it is a recently acquired property of *L. plantarum* CMPG5300 through horizontal gene transfer. This transfer might have promoted the adaptation of strain CMPG5300 to the vaginal niche, as reflected by our functional assays.

The first niche-related function identified for Msl is its capacity to promote self-aggregation of *L. plantarum* CMPG5300. Self-aggregation is postulated to increase the colonization potential of lactobacilli in environments with short residence times^22^. Auto-aggregation has been documented before for other vaginal *Lactobacillus* strains^4^,^23^ but without details on the molecules involved. We previously observed a loss of auto-aggregation in the sortase A (*srtA*) mutant^24^. The fact that the *msl* mutant of *L. plantarum* CMPG5300 is also clearly impaired in auto-aggregation now shows a role for the sortase-dependent Msl lectin in the auto-aggregation of this strain. Furthermore, we could substantiate a role for the Msl sortase-dependent protein (SDP) in adhesion of this bacterium to the vaginal VK2/E6E7 cell line. This is in agreement with our previous observations that showed a significant decrease in adhesion of the sortase mutant to the VK2/E6E7 cell line^24^. Even though this phenotype was restored in the complemented strain, the auto-aggregating and the adhesive capacities were observed to be reduced when compared with the wild-type. This is in accordance with the results of the complemented *srtA* strain CMPG5378 (Malik *et al.*^24^) where the phenotype could not be restored to the wild type level. This could partly be due to the copy number effect where the copy number of Cmpg5300.05_29 on the endogenous plasmid of CMPG5300 is different than the one on CMPG11202. Former work on the adhesive factors of vaginal *Lactobacillus* strains has indicated the involvement of glycoproteins in *Lactobacillus acidophilus* and *Lactobacillus gasseri*, carbohydrates in *Lactobacillus jensenii*^25^ and a non-secretory substance in *Lactobacillus crispatus* CTV-05^26^. However, it remains to be determined whether a related lectin mediates the adhesion in these other vaginal strains as well. For the strain *L. plantarum* CMPG5300, further functional characterization of the *msl* mutant also substantiates a role for the Msl lectin in biofilm formation. This phenotype is probably directly linked to the role of Msl in auto-aggregation as a first step in biofilm formation.

High-mannose N-type glycans are found on the glycoproteins of HIV-1 especially on its glycoprotein gp120^27^. The use of CBAs is thus currently explored as a novel microbicidal strategy to prevent HIV infection in the vagina^28^. We observed that Msl has the capacity to interact with the high-mannose envelope glycoprotein of the pathogen HIV. While wild-type *L. plantarum* CMPG5300 clearly bound to the HIV glycoprotein gp120 (based on ELISA), the *msl* mutant significantly lost this binding capacity. In addition, the purified lectin domain exhibited a strong gp120 binding capacity, as determined by SPR analyses, which could be blocked in the presence of an oligomer of mannose. Of note, the mannosylation of gp120 closely resembles the glycans to which Msl shows the strongest binding (Manα1-2Manα1-6(Manα1-3)Manα1-6(Manα1-2Manα1-2Manα1-3)Manβ1-4GlcNAcβ1-4GlcNAcβ-Sp12). Some mannose-binding plant lectins are known to have anti-HIV activity by virtue of their specific binding to the highly mannosylated glycoprotein gp120^27^. Moreover, mannose-binding lectin (MBL) detected in vaginal secretions of women was found to be antimicrobial in nature and its deficiency is associated with the recurrence of infections by *C. albicans*^29^. The present study characterizes an HIV-1 gp120 binding lectin from a *Lactobacillus* strain. However, we were not able to observe anti-HIV-1 activity *in vitro* in the standard cellular assays at the tested concentrations (up to 200 μg/ml) of the lectin domain. This may indicate that the Msl lectin domain binds to gp120 in a non-neutralizing manner like some antibodies, as documented previously^30^. Alternatively, the lectin may be quickly inactivated in the cell culture due to the presence of proteases in the bovine serum-containing medium or to the presence of serum sugars that bind (neutralize) the bacterial lectin. Since the Msl lectin is normally presented on live *L. plantarum* CMPG5300 cells and this configuration is probably important *in vivo* for pathogen interaction of these lactobacilli in the vaginal niche, we believe that the lack of efficacy observed in these *in vitro* assays should not exclude further exploration of the trapping of HIV by lectins/live lactobacilli. Such trapping could eventually eliminate the virus from the vaginal environment by the vaginal fluid *in vivo* since lectin-mediated binding of vaginal lactobacilli to pathogens could expose the latter to the antimicrobial compounds secreted by lactobacilli, including lactic acid. However, experimental substantiation of such *in vivo* activity for specific lectins and lactobacilli is severely hampered by the difficulty of animal models for HIV^31^, although some progress is being made^32^.

Since it can be envisaged that it is more difficult to inhibit invasion of small viral particles compared to larger bacteria, we subsequently focused on the possible role of Cmpg5300.05_29 in preventing bacterial infections by investigating its role in inhibiting bacterial biofilms. Recurrent biofilms are a major problem in urogenital infections^33^. Our results show that the lectin domain of Msl has a remarkable capacity to prevent UPEC and *S.* Typhimurium biofilm formation, as well as moderately affect biofilms of *S. aureus* strains. Previous studies have reported the ability of probiotic lactobacilli to interfere with pathogenic biofilms^34^,^35^,^36^, but without identifying the underlying mode of action. Our findings show the inhibitory capacity of a bacterial, and more specifically a *Lactobacillus* lectin, against bacterial pathogens. The localization of Msl within pathogenic biofilms as observed after FITC-labeling suggests that the lectin interacts with components of the biofilm matrix, composed of extracellular polymeric substances, including polysaccharides, proteins such as fimbriae and lectins, DNA and lipids^37^ and interferes with stabilization of the biofilm. The composition of the biofilm matrix varies among strains, which may also explain the observed species-specific activity of the lectin against various species. For example, both *S.* Typhimurium and *E. coli* biofilm matrices contain the polysaccharides cellulose (β-1, 4-D-glucose polymer) and colanic acid (heteropolysaccharide of glucose, galactose, fucose and glucuronic acid)^38^,^39^, which can be a target for Msl. The substantiation of biofilm-inhibiting effect using *in vivo* studies including competitive exclusion is worth further exploration, given the prevalence of problems associated with biofilms and the increased resistance of various bacteria against antibiotics^40^. The Msl lectin, by itself or when present on live lactobacilli, could then have a promising potential for local application for exclusion of high-mannose containing pathogens.

This study is thus of relevance for the use of whole bacterial cells as prophylactic probiotics, since the lectins promote the potential colonization capacity of the strains in the human vagina. The enhanced colonization of these probiotics could in turn prevent adhesion and colonization by pathogens causing vaginal infections. Alternatively, the isolated lectin could be used in therapeutic mode for urogenital as well as gastrointestinal infections, for instance, in synergy with antibiotics to disrupt biofilms that are otherwise difficult to treat only with antibiotics.

## Material and Methods

### Bacterial strains and culture conditions

*L. plantarum* CMPG5300 and its mutant derivatives (Table 2) were routinely grown without agitation in Man Rogosa Sharpe (MRS) (Difco) at 37 °C. For cloning purpose, *E. coli* TOP10 and BL21/DE3 were grown with shaking at 37 °C in Luria Bertani (LB) medium. When required, antibiotics were added at the following final concentrations: chloramphenicol, 20 μg/ml for *L. plantarum* and 10 μg/ml for *E. coli*; erythromycin, 10 μg/ml for *L. plantarum* and 250 μg/ml for *E. coli*; ampicillin 100 μg/ml for *E. coli* and kanamycin 50 μg/ml for *E. coli*.

*E. coli* UTI89, *P. aeruginosa* PA14, *S*. Typhimurium ATCC14028, *S. aureus* Rosenbach and SH1000 were grown routinely in LB medium with aeration at 37 °C. *C. albicans* SC5314 and *S. cerevisiae* BY4741 were grown in yeast-extract peptone dextrose (YPD) medium (2% peptone, 1% yeast extract, 2% glucose) under aerobic conditions at 37 °C.

### Identification of *cmpg5300.05_29* and Southern hybridization

The genome mining of CMPG5300 and its alignment with *L. plantarum* WCFS1 in our previous study^11^ led to the identification of *cmpg5300.05_29* as a potential lectin-encoding gene of CMPG5300. This gene occurred on contig 54 and is potentially plasmid-encoded. To obtain the complete gene sequence, the gaps were closed by using the primers mentioned in Table S1. Furthermore, as the contig 54 containing the *cmpg5300.05_29* gene sequence did not align with the *msa* gene of *L. plantarum* WCFS1 (*lp_1229*), efforts were made to identify whether the gene was present on the chromosomal DNA or plasmid DNA. For this purpose, Southern hybridization^41^ was performed using the genomic DNA and plasmid DNA isolated from CMPG5300 (Malik *et al.*^24^). The primers used for constructing the probes are listed in supplementary information Table S1.

### Construction of *cmpg5300.05_29* knock-out mutant

In order to study the role of Cmpg5300.05_29, its gene was knocked out using double homologous recombination using a cre-lox strategy^42^ as described previously for the sortase gene *srtA* in *L. plantarum* CMPG5300^24^. The corresponding plasmids and primers used are mentioned in Table 2 and Table S1, respectively. The mutant, CMPG11201, was selected on the basis of resistance against chloramphenicol and checked by PCR and Southern hybridization. For complementation, *cmpg5300.05_29* was amplified using the primers PRO 8367 and PRO 8368 and ligated downstream of the *dlt* promoter in the vector pCMPG10208 resulting in pCMPG11202. CMPG11201 was complemented with *cmpg5300.05_29* by electroporation of pCMPG11202 resulting in strain CMPG11202. To validate gene complementation, qRT-PCR was performed as done for CMPG5376 (Malik *et al.*^24^) using primers mentioned in Table S1.

### Heterologous expression and purification of lectin domain of Cmpg5300.05_29

The recombinant *E. coli* (BL21/DE3) (Table 2) expressing the lectin domains of Cmpg5300.05_29 (plasmid pCMPG11209) and Msa of *L. plantarum* WCFS1 (pCMPG11210) were grown overnight in LB with 50 μg/ml kanamycin. Each culture was diluted 100-fold in 2 liter LB with kanamycin and grown for 2 to 3 h at 37 °C under agitation until an optical density (OD) (595 nm) between 0.3 and 0.4 was reached. Then the production of recombinant lectin domain was induced with 1 mM isopropyl β-D-1-thiogalactopyranoside (IPTG) (Sigma-Aldrich). The next day, the pellets were suspended in 20 ml of non-denaturing lysis buffer (Sodium dihydrogen phosphate 50 mM, Sodium chloride 300 mM, Imidazole 10 mM, pH 8) per liter of original culture and incubated for 30 min at room temperature (RT) with gentle swirling. Subsequently the cell lysate was sonicated during 4 min in cycles of 30 sec on and 30 sec off (amplitude 18%) to release the soluble recombinant lectin domain from the cells. Subsequently, affinity chromatography was used to purify the lectin domains. Hereto, the lysate was first filtered with 0.2 μM filters and the filtered lysate was run through a HisTrapTM HP column (GE Healthcare). Different fractions were collected and sample purity was analyzed through sodium dodecyl sulfate-polyacrylamide gel electrophoresis (SDS-PAGE). The lectin domains were further purified from the eluted sample using size exclusion chromatography, using a HighloadTM 16/60 column packed with a matrix of SuperdexTM prep grade (GE Healthcare). Fractions containing the purified lectin domains were collected, analyzed using SDS-PAGE and Western Blotting (with primary mouse monoclonal anti-His6 antibodies), pooled together and concentrated.

### *In vitro* adhesion and biofilm formation

To assess the role of Cmpg5300.05_29 in adhesion of the strain to the vaginal epithelial cell line VK2/E6E7 and in biofilm formation on polystyrene, CMPG5300 and its mutant derivatives were analyzed using the adhesion and biofilm assays described previously (Malik *et al.*^24^).

Briefly the adherence of *L. plantarum* CMPG5300 strains to the epithelial cells was examined by adding 1.5 ml of DMEM containing 10^7^ CFU/ml. After incubation at 37 °C for 1 h, epithelial cells were washed twice with pre-warmed PBS. Subsequently, 100 μl of trypsin-EDTA (1x) (Invitrogen) was added to each well and incubated for 10 min at 37 °C. Finally, 900 μl of PBS was added, mixed and serial dilutions were plated out on MRS plates. Alternatively, a fluorescence assay was performed as described previously^19^ with minor modifications. The FITC labelled lectin domain of Cmpg5300.05_29 was suspended in the DMEM medium in the absence of serum and antibiotics, and incubated for 1 h with the monolayers of VK2/E6E7 cells grown on 13-mm coverslips. After incubation, the cells were washed three times with PBS, and fixed with 4% paraformaldehyde for 10 min. Slides were examined with a Zeiss Axio Imager Z1 microscope using an EC Plan Neofluar (X40 magnification/0.3 numerical aperture) objective (excitation 488 nm, emission 511 nm). Pictures were acquired with an AxioCam MRm and the AxioVision.

### Mannose-dependent *S. cerevisiae* and *C. albicans* agglutination assay

A yeast agglutination assay was performed using the protocol described previously (Pretzer *et al.*^12^) but with minor modifications. Briefly, overnight-grown cultures of *Lactobacillus* strains were washed, suspended in phosphate buffered saline (PBS, pH 7.2) to a final concentration of 1 × 10^10^ CFUs/ml. Similarly, overnight cultures of *S. cerevisiae* or *C. albicans* cells were washed and suspended in PBS so as to a make a 1% w/v cell suspension. For agglutination, 25 μl of bacterial suspension was added to 25 μl of PBS followed by 50 μl of yeast cells suspension in a 96-well U-bottom well sterile plate (Greiner bio-one). The mixtures were incubated for 10 min at RT with gentle shaking. To study inhibition, 25 μl of methyl-α-D-mannopyranoside (50 mM; Sigma-Aldrich) was added to the bacterial suspension instead of PBS. To examine agglutination, the mixtures were spotted on a slide and viewed under a phase-contrast microscope (400-fold magnification, Zeiss Axio Imager Z1 microscope equipped with an AxioCam MRm Rev.3 monochrome digital camera). Alternatively *C. albicans* and *S. cerevisiae* were incubated with 200 μg/ml of fluorescein isothiocyanate (FITC)-labeled lectin domain of Cmpg5300.05_29 alone and in combination with 25 μl of methyl-α-D-mannopyranoside (50 mM; Sigma-Aldrich). As a control, a lectin L-type domain of lectin-like protein 2 (Llp2) from *L. rhamnosus* GG was used.

### ELISA-based assay for assessing sugar binding

For a quantitative analysis of mannan binding, an ELISA-based assay (Velez *et al.*^43^) was used with minor modifications. The microtiter (X50 Immulon 4HBX 96 well plates, Fischer Scientific) plate was coated with 10 μg mannan from *S. cerevisiae* (Sigma Aldrich NV) or Bovine Serum Albumin (BSA) (Sigma Aldrich NV) (negative control) per well prepared in carbonate-bicarbonate buffer (pH 9.6) and incubated overnight at 4 °C. Overnight cultures of *Lactobacillus* strains were washed twice with PBS and suspended in 0.1 M carbonate buffer (pH 8.2) up to a final concentration of 1 × 10^10^ CFUs/ml. For labeling the cells, biotin *N*-hydroxysuccinimide ester (Sigma) was added to the cells at a concentration of 100 μg/ml and the cells were incubated at RT for 1 h with gentle swirling. Subsequently, the wells were washed with distilled water and blocked with blocking buffer (TBSt20 with 0.5% blocking agent) for 2 h at RT. Simultaneously, the biotin-labelled cells were washed thrice with PBS and suspended in PBS so as to make a working concentration of 2.5 × 10^9^ CFUs/ml. The cells were added to the pre-coated wells which were then washed with PBS and incubated for 1 h at 37 °C. Subsequently, the plate was washed with PBST (PBS with 0.025% Tween 20) to remove unbound cells. Color was developed by adding Alkaline Phosphatase (AP)-Strep (1:5000 in blocking buffer) in each well for 1 h at 37 °C. The plate was washed and 4-nitrophenyl phosphate (NPP) disodium salt (Sigma; 1 mg/ml in carbonate bicarbonate buffer) was added as the substrate. Color was allowed to develop for 15–30 min at RT and the absorbance was read at 405 nm. The experiments were performed at least three times with six technical repeats each.

### Pull-down sugar binding assays using Sepharose beads

Sepharose^®^ 6B beads (Sigma-Aldrich) were coated with 20% D-glucose, GlcNac, D-mannose, D-fucose and mannan of *S. cerevisiae*, as previously described^44^,^45^ with minor modifications. For the sugar-binding assay, 25 μl of each set of coated functionalized beads was washed with binding buffer as previously described^45^. 1 ml of binding buffer containing 50 μg of the purified lectin domain of Cmpg5300.05_29 was added to each set of beads. Hereafter, the mixtures were incubated for 2 h at 4 °C. The beads were washed twice with 1 ml of wash buffer and bound samples of lectin domain were eluted by boiling the beads in SDS-PAGE loading buffer (Fermentas, Life Sciences) for 10 min at 95 °C. The eluates were resolved by SDS-PAGE through 12% polyacrylamide gels (Life Sciences), which were stained with Sypro^®^ Ruby protein gel stain (Invitrogen) and scanned using the Typhoon scanner (GE Healthcare Life Sciences).

### Glycan array analysis

To test a larger number of glycosylated substrates, the purified lectin domain was subsequently sent for glycan array screening at the Consortium for Functional Glycomics (CFG). The mammalian glycan array version 5.2 was used to determine the exact sugar specificity of the lectin domain. The array consists of 609 glycan targets of natural and synthetic mammalian glycans with amino linkers and is printed onto N-hydroxysuccinimide (NHS)-activated glass microscope slides (SCHOTT Nexterion), forming covalent amide linkages. The purified sample (lectin domain of Cmpg5300.05_29) was labeled with FITC by using FluoReporter^®^ FITC Protein Labeling Kit (Life Technologies) according to the producer’s manual. Two different concentrations of FITC labeled protein, namely 20 μg/ml and 200 μg/ml, were used to determine the exact sugar-binding capacity. The experiment was then performed by the Consortium for Functional Glycomics (CFG, www.functionalglycomics.org).

### HIV-1 gp120 and gp41 binding assay via ELISA and SPR analysis

To determine the role of Cmpg5300.05_29 in binding to HIV-1 envelope glycoprotein gp120 and gp41, the cells of *L. plantarum* CMPG5300 and *msl* mutant strain CMPG11201 were labeled with biotin, incubated with 2 μg/ml immobilized HIV-1 gp120 or gp41 (ImmunoDiagnostics Inc., Woburn, MA) (produced by CHO cell cultures) and assessed for binding using the ELISA-based method described above for mannan.

Furthermore, binding of the lectin domain was assessed by SPR analysis using the method described previously (Balzarini *et al.*^16^). Briefly, recombinant gp120 protein from HIV-1 strain IIIB and recombinant gp41 protein from HIV-1 (HXB2 strain) (Acris Antibodies GmbH, Herford, Germany) (produced by *Pichia pastoris*) were covalently immobilized on the carboxymethylated dextran matrix of a CM4 sensor chip in 10 mM sodium acetate, pH 5.0, using standard amine coupling chemistry at final densities of 115 RUs and 45 RUs, respectively. In another set of experiments, binding of the lectin domain to gp120 and gp41 was subjected to a detailed kinetic characterization. A 1:1 fit was applied to obtain the association rate constant (*ka*), the dissociation rate constant (*kd*), and the apparent kinetic rate constant (*K*_*D*_). All interaction studies were performed at 25 °C on a Biacore T200 instrument (GE Healthcare, Uppsala, Sweden) in HBS-P (10 mM HEPES, 150 mM NaCl and 0.05% surfactant P20; pH 7.4) containing10 mM CaCl_2_. The CM4 sensor chip surface was regenerated with a single injection of 50 mM NaOH.

### HIV-1 inhibition assays

The inhibitory activity of purified lectin domain against HIV-1(III_B_)- and HIV-2-induced cytopathicity in C8166 and Raji DCSIGN cell cultures was examined in microtiter 96-well plates containing ~3 × 10^5^ cells/ml infected with 100 CCID_50_ of HIV per ml and appropriate dilutions of the test samples. After 4–5 days of incubation at 37 °C, C8166-induced giant (syncytium) cell formation was examined microscopically and the 50% effective concentrations (EC_50_) determined.

### Antimicrobial assays for interference with pathogen grown in suspension

In order to verify whether the lectin domain of Cmpg5300.05_29 affects the growth of pathogens, bioscreen experiments were performed. Overnight cultures of *S. aureus* SH1000, *S. aureus* Rosenbach, *E. coli* UTI89, and *S.* Typhimurium ATCC14028 were diluted 200-fold in Tryptic Soy Broth (TSB) medium and 200 μl of cell suspensions were added to sterile wells of 100-well microtiter plates (Honeycomb, Oy Growth Curves Ab Ltd). The purified lectin domain of Cmpg5300.05_29 was added at concentrations of 50 μg/ml and 200 μg/ml. These were incubated at 37 °C for 3 days under continuous agitation in a Bioscreen (Oy Growth Curves Ab Ltd), which measured the OD at 600 nm every 10 min to monitor growth. TSB was used as blank. Each strain and concentration of the sample containing lectin domain was tested in triplicate.

### Antimicrobial assays for interference with pathogen growth in biofilms

The static peg biofilm assays were performed as described previously with minor modifications^46^,^47^. Hereto, *E. coli* UTI89, *P. aeruginosa* PA14, *S. aureus* SH1000, *S. aureus* Rosenbach and *S.* Typhimurium ATCC14028 were grown in the presence of 50 or 200 μg/ml of lectin domain of Cmpg5300.05_29. After 48 h of incubation, biofilm formation was quantified by coloring with crystal violet (0.1% w/v in 5% methanol, 5% isopropanol and 90% PBS). For each strain and concentration tested, the experiment was performed at least three times with eight technical repeats. Alternatively, the lectin domain of Cmpg5300.05_29 was added after 1.5 h (the initial adhesion phase of the tested pathogens). In a third version of the assay, the sample was added with fresh medium after 24 h to investigate if the lectin domain could disrupt an already established biofilm.

For the visualization of *E. coli* UTI89 and *S.* Typhimurium biofilms the FITC- labeled lectin domain of Cmpg5300.05_29 was added at the onset of the biofilm formation (t = 0 h) at 50 μg/ml concentration and the biofilms were grown for 48 h. Microscopic epifluorescent imaging was performed using a Zeiss Axio Imager Z1 microscope with an EC Plan Neofluar (X40 magnification/0.3 numerical aperture) objective (excitation 488 nm, emission 511 nm). Pictures were acquired with an AxioCam MRm and the AxioVision software.

### Statistical analysis

To determine significant differences the unequal variance t-test was applied. A P-value below 0.05 was considered as statistically significant.

## Additional Information

**How to cite this article**: Malik, S. *et al.* High mannose-specific lectin Msl mediates key interactions of the vaginal *Lactobacillus plantarum* isolate CMPG5300. *Sci. Rep.* **6**, 37339; doi: 10.1038/srep37339 (2016).

**Publisher’s note**: Springer Nature remains neutral with regard to jurisdictional claims in published maps and institutional affiliations.

## Supplementary Material

Supplementary Information

## Figures and Tables

**Table 1:** Kinetic data for the interaction of the lectin domain of *L. plantarum* CMPG5300 with HIV-1 gp120 and gp41.

	HIV-1 gp120			HIV-1 gp41		
	ka (1/Ms)	kd (1/s)	KD (M)	ka (1/Ms)	kd (1/s)	KD (M)
HHA	1.66E + 06	2.19E − 03	1.32E − 09	2.75E + 06	2.29E − 03	8.36E − 10
Lectin domain of Msa	2.62E + 03	1.64E − 02	6.26E − 06	7.15E + 03	2.36E − 02	3.30E − 06
Lectin domain of Msl	3.05E + 04	5.64E − 02	1.85E − 06	2.29E + 04	2.88E − 02	1.62E − 06

ka: association rate constant, kd: dissociation rate constant and KD: the binding affinity values.

**Table 2:** Strains and plasmids used in this study.

Name of the species	Name of the strain	Relevant genotype/description	Reference/source
**Bacterial/yeast strains**			
*L. plantarum*	CMPG5300	Wild type, human vaginal isolate	Malik *et al.*^24^
	CMPG11201	*Δcmpg5300.05_29*::Cm^r^	This study
	CMPG11202	CMPG11201 complemented with *cmpg5300.05_29*	This study
	WCFS1	Sequenced wild-type strain; single colony isolate of NCIMB 8826 from human saliva	48
*E. coli*	TOP10F	F’ (*lacIq*, Tnr) *mcr*A_(*mrr hsd*RMS-*mcr*BC) 80LacZ_lacX74 deoR recA1 araD139_(araleu)	Invitrogen
	BL21/DE3	*E. coli* B F– *dcm ompT hsdS*(r_B_^−^ m_B_^−^) *gal* λ(DE3)	Invitrogen
	UTI89	Wild type, clinical isolate	49
*P. aeruginosa*	PA14	Wild type, human isolate	50
*S. cerevisiae*	BY 4741	MATa; his3Δ 1; leu2Δ 0; met15Δ 0; ura3Δ 0	51
*C. albicans*	SC5314 (ATCC MYA-2876)	Wild type, human clinical isolate	52
*S. aureus*	SH1000	*rsbU* positive derivative of *S. aureus* 8325-4	53
	Rosenbach (ATCC 33591)	Wild type, clinical isolate	ATCC
*S. enterica* serovar Typhimurium	ATCC 14028	Wild type, isolated from chicken tissue	ATCC^54^
	ATCC 14028 carrying pFPV25.1	Mutant constitutively expressing GFP	46
	**Host**	**Relevant description**	**Reference/source**
**Plasmids**			
pNZ5319	*E. coli*	pNZ5318 derivative for multiple gene replacements in gram-positive bacteria Cm^r^ Ery^r^	Lambert *et al.*^42^
pCMPG10208	*E. coli*	pLAB1301 derivative driven by *dlt* promoter, Ap^r^, Ery^r^	Unpublished results
pCMPG11201	*E. coli*	pNZ5319 derivative containing homologous regions up- and downstream of *cmpg5300.05_29*, Cm^r^ Ery^r^	This study
pCMPG11202	*E. coli*	pCMPG10208 derivative containing *cmpg5300.05_29* of CMPG5300, Ap^r^, Ery^r^	This study
pET28 a+	*E. coli*	Kan^r^, T7 lac, N and C-terminal His Tag	Novagen
pCMPG11209	*E. coli*	pET28a+ derivative containing the lectin domain of *cmpg5300.05_29* of CMPG5300, Kan^r^	This study
pCMPG11210	*E. coli*	pET28a+ derivative containing the lectin domain of *msa* gene of *L. plantarum* WCFS1, Kan^r^	This study
