# Supplementary material for: High mannose-specific lectin Msl mediates key interactions of the vaginal Lactobacillus plantarum isolate CMPG5300
Source: Sci Rep. 2016 Nov 17;6:37339. doi: 10.1038/srep37339 (PMC5112522; doi:10.1038/srep37339)
Supplement: Supplementary Information [file srep37339-s1.pdf]

High mannose specific lectin Msl mediates key interactions of the vaginal *Lactobacillus plantarum* isolate CMPG5300

Shweta Malik[1, 2#], Mariya I. Petrova[1, 2#], Nicole C.E. Imholz[1, 2], Tine L.A. Verhoeven[1], Sam Noppen[3], Els J.M. Van Damme[4], Sandra Liekens[3], Jan Balzarini[3], Dominique Schols[3], Jos Vanderleyden[1], Sarah Lebeer[1, 2*]

Figure S1. (**a**) Anti-biofilm activity of ConA, HHA and D-mannose against *E. coli* UTI89 and *S.* Typhimurium ATCC14028. The lectins (ConA and HHA) or D-mannose were added at 50 µg/ml concentration at the onset of the biofilm (t=0h). (**b**) Anti-biofilm activity of the lectin domain of Cmpg5300.05_29 against *P. aeruginosa* PA14 when added at 50 µg/ml and 200 µg/ml.

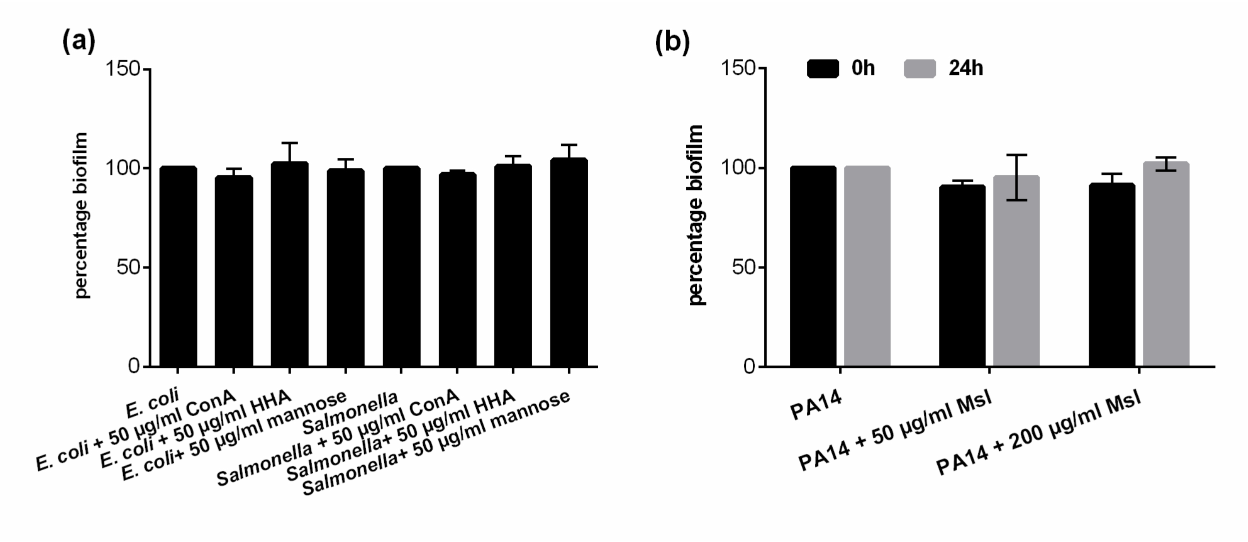

Table S1. List of primers used in the study.

**Primer name**	**Sequence (5' – 3')**	**Restriction site**	**Purpose**
**For closing the gap:**			
PRO7245	CCCAATAGCCTTAATAAACCGGGA		To amplify the 5' end of the upstream region of *cmpg5300.05_29* of CMPG5300
PRO7246	CAGCATTACCAATATCTGTGG		To amplify the 3' end of *cmpg5300.05_29* of CMPG5300 for gap closing and qRT-PCR
PRO7247	TTGGATCTGCCGTATATCC		To amplify the 3' end of *cmpg5300.05_29* of CMPG5300
PRO7248	CCACAGATATTGGTAATGCTG		To amplify the 5' end of *cmpg5300.05_29* of CMPG5300
PRO7447	GGGTCAACCGGCTTTG		To amplify the 3' end of *cmpg5300.05_29* of CMPG5300
PRO7448	CAACAACGTATTACTCAACTAAAGC		To amplify the 5' end of *cmpg5300.05_29* of CMPG5300
PRO7798	GCTTTGTTGGAGCATCCTTGG		To amplify the 3' end of *cmpg5300.05_29* of CMPG5300
PRO7800	GCGTGAAGTCTCAACTGATGCAG		To amplify the 5' end of *cmpg5300.05_29* of CMPG5300
PRO7522	GCTAGTGATCTTGACTTTACAG		To amplify the 5' end of *cmpg5300.05_29* of CMPG5300
PRO7334	GTCAAGATCACTAGCGTCACC		To amplify the 3' end of *cmpg5300.05_29* of CMPG5300
PRO7672	ATGAATTCCAACTGATGCGGTGACA		To amplify the 5' end of *cmpg5300.05_29* of CMPG5300
PRO7964	GATCACTGGAGTCACCACTCACAG		To amplify the 3' end of *cmpg5300.05_29* of CMPG5300
**For cloning:**			
PRO 7036	ATCTCGAGTCGGCGTATTGTCAATTAAG	*Xho1*	To amplify the 5' end of HR1 of *cmpg5300.05_29*
PRO 7037	ATGTTTAAACAAACTAAGATCCAGTTTGAGAGCT	*Pme1*	To amplify the 3' end of HR1 of *cmpg5300.05_29* and to amplify the 3' end of the transcriptional regulator upstream of the *cmpg5300.05_29* to use the region as a probe
PRO 7038	ATGAGCTCCCGGTTTATTAAGGCTATTGGG	*EclI3611*	To amplify the 5' end of HR2 of *cmpg5300.05_29*
PRO 7039	ATAGATCTTCTTACCCATTAAAGATTCAAACGT	*BglII*	To amplify the 3' end of HR2 of *cmpg5300.05_29*
PRO 7285	AGCTCTCAAACTGGATCTTAGTTT		5' end of flanking region of *cmpg5300.05_29* to check gene replacement

PRO 7245	CCCAATAGCCTTAATAAACCGGGA		3’ end of flanking region of *cmpg5300.05_29* to check gene replacement
cat96F	TCAAATACAGCTTTTAGAACTGG		5’ end of cat cassette pNZ5319 (Kleerebezem et al, 2007)
cat96R	ACCATCAAAAATTGTATAAAGTGGC		3’ end of cat cassette pNZ5319 (Kleerebezem et al, 2007)
EryintF	CGATACCGTTTACGAAATTGG		5’ end of ery cassette pNZ5319 (Kleerebezem et al, 2007)
EryintR	CTTGCTCATAAGTAACGGTAC		3’ end of ery cassette pNZ5319 (Kleerebezem et al, 2007)
PRO 7838	GGAAGGCACGTTAGGAATCATTACC		5’ end of pNZ5319 to check gene replacement
PRO 7839	GAGATCTTCTTCACGTTACTAAAGGG		3’ end of pNZ5319 to check gene replacement
PRO 8367	ATCCCGGGCCCAATAGCCTTAATAAACCGGGA	*Xma1*	To amplify the 5’ end of *cmpg5300.05_29* for complementation
PRO 8368	ATGAGCTCCCTATTCTTTGCGCTGCC	*Sac1*	To amplify the 3’ end of *cmpg5300.05_29* for complementation
pseu383	CCCCCTGACGAGCATCACAA		3’ end of pLAB1301 to check insertion in the MCS
pseu384	GCGTCAGACCCCGTAGAAAA		5’ end of pLAB1301 to check insertion in the MCS
PRO 0744	TCAAGTTTGTTAAGATGGAT		To amplify the 5’ end of region of the *dlt* promoter of *L. rhamnosus* GG and 5’ upstream region of plasmid pCMPG10208 to check plasmid insertion
PRO 0745	GACCCGATCAATCGCTGTAATAAC		To amplify the 3’ end of region of the *dlt* promoter *of L. rhamnosus* GG
**For Southern hybridization:**			
PRO 7189	CCCAAGGGTTTTAACAGACCGATC		To amplify the 5’ end of the transcriptional regulator upstream of the *cmpg5300.05_29* to use the region as a probe
**For heterologous expression:**			
PRO 7903	TGGCTAGTCGACAGAGTAAGGCTTTGGCGACTGATAAA	*Sal1*	To amplify the 5’ end of the lectin domain of *cmpg5300.05_29* of CMPG5300 for heterologous expression and qRT-PCR
S&P-000154	TGGCTAGCGGCCGCTTAGCAATTTGATTACCTTTTGTATCAACATA	*Not1*	To amplify the 3’ end of the lectin domain of *cmpg5300.05_29* of CMPG5300
PRO 7906	TGGCTAGTCGACAGGAAGCGGCCTTGACTCAT	*Sal1*	To amplify the 5’ end of the lectin domain of *msa* gene of *L. plantarum* WCFS1 for heterologous expression and also for *lp_1229* probe

			construction
PRO 7907	TGGCTAGCGGCCGCTTACATTC ACAGTCGCTGCTTCTTG	*Not1*	To amplify the 3’ end of the lectin domain of *msa* gene of *L. plantarum* WCFS1 for heterologous expression and also for *lp_1229* probe construction
S&P-00044	TGGCAGCAGCCAACTCAGCT		3’ end of pET28a+ to check insertion in the MCS
S&P-00045	TATAGGCGCCAGCAACCGCA		5’ end of pET28a+ to check insertion in the MCS
**For qPCR (to check complementation):**			
PRO 8696	ATCTCGAGGCACTGCTACTAC TTGTACTAGTCG		To amplify the 3’ end of *cmpg5300.05_29* for qRT-PCR
PRO 7446	GTAGCAATAGGATATACGGCA G		To amplify the 5’ end of *cmpg5300.05_29* for qRT-PCR
PRO 7796	GCTTTAGTTGAGTAATACGTTG TTG		To amplify the 3’ end of *cmpg5300.05_29* for qRT-PCR
PRO 7696	ATGTCGACCATTAAACGGATC AGCCAC		To amplify the 5’ end of *cmpg5300.05_29* for qRT-PCR

Table S2 List of glycans printed on the glycan array

Chart Number	Glycan structures
1	Galα-Sp8
2	Glcα-Sp8
3	Manα-Sp8
4	GalNAcα-Sp8
5	GalNAcα-Sp15
6	Fucα-Sp8
7	Fucα-Sp9
8	Rhaα-Sp8
9	Neu5Acα-Sp8
10	Neu5Acα-Sp11
11	Neu5Acβ-Sp8
12	Galβ-Sp8
13	Glcβ-Sp8
14	Manβ-Sp8
15	GalNAcβ-Sp8
16	GlcNAcβ-Sp0
17	GlcNAcβ-Sp8
18	GlcN(Gc)β-Sp8
19	Galβ1-4GlcNAcβ1-6(Galβ1-4GlcNAcβ1-3)GalNAcα-Sp8
20	Galβ1-4GlcNAcβ1-6(Galβ1-4GlcNAcβ1-3)GalNAc-Sp14
21	GlcNAcβ1-6(GlcNAcβ1-4)(GlcNAcβ1-3)GlcNAc-Sp8
22	6S(3S)Galβ1-4(6S)GlcNAcβ-Sp0
23	6S(3S)Galβ1-4GlcNAcβ-Sp0
24	(3S)Galβ1-4(Fucα1-3)(6S)Glc-Sp0
25	(3S)Galβ1-4Glcβ-Sp8
26	(3S)Galβ1-4(6S)Glcβ-Sp0
27	(3S)Galβ1-4(6S)Glcβ-Sp8
28	(3S)Galβ1-3(Fucα1-4)GlcNAcβ-Sp8
29	(3S)Galβ1-3GalNAcα-Sp8
30	(3S)Galβ1-3GlcNAcβ-Sp0
31	(3S)Galβ1-3GlcNAcβ-Sp8
32	(3S)Galβ1-4(Fucα1-3)GlcNAc-Sp0
33	(3S)Galβ1-4(Fucα1-3)GlcNAc-Sp8
34	(3S)Galβ1-4(6S)GlcNAcβ-Sp0
35	(3S)Galβ1-4(6S)GlcNAcβ-Sp8
36	(3S)Galβ1-4GlcNAcβ-Sp0
37	(3S)Galβ1-4GlcNAcβ-Sp8
38	(3S)Galβ-Sp8
39	(6S)(4S)Galβ1-4GlcNAcβ-Sp0
40	(4S)Galβ1-4GlcNAcβ-Sp8
41	(6P)Manα-Sp8
42	(6S)Galβ1-4Glcβ-Sp0

43	(6S)Galβ1-4Glcβ-Sp8
44	(6S)Galβ1-4GlcNAcβ-Sp8
45	(6S)Galβ1-4(6S)Glcβ-Sp8
46	Neu5Acα2-3(6S)Galβ1-4GlcNAcβ-Sp8
47	(6S)GlcNAcβ-Sp8
48	Neu5,9Ac2α-Sp8
49	Neu5,9Ac2α2-6Galβ1-4GlcNAcβ-Sp8
50	Manα1-6(Manα1-3)Manb1-4GlcNAcβ1-4GlcNAcβ-Sp12
51	Manα1-6(Manα1-3)Manβ1-4GlcNAcβ1-4GlcNAcβ-Sp13
52	GlcNAcβ1-2Manα1-6(GlcNAcβ1-2Manα1-3)Manβ1-4GlcNAcβ1-4GlcNAcβ-Sp12
53	GlcNAcβ1-2Manα1-6(GlcNAcβ1-2Manα1-3)Manβ1-4GlcNAcβ1-4GlcNAcβ-Sp13
54	Galβ1-4GlcNAcβ1-2Manα1-6(Galβ1-4GlcNAcβ1-2Manα1-3)Manβ1-4GlcNAcβ1-4GlcNAcβ-Sp12
55	Neu5Aca2-6Galβ1-4GlcNAcβ1-2Manα1-6(Neu5Acα2-6Galb1-4GlcNAcβ1-2Manα1-3)Manβ1-4GlcNAcβ1-4GlcNAcβ-Sp12
56	Neu5Aca2-6Galβ1-4GlcNAcβ1-2Mana1-6(Neu5Aca2-6Galβ1-4GlcNAcβ1-2Manα1-3)Manβ1-4GlcNAcβ1-4GlcNAcβ-Sp21
57	Neu5Aca2-6Galβ1-4GlcNAcβ1-2Manα1-6(Neu5Aca2-6Galβ1-4GlcNAcβ1-2Manα1-3)Manβ1-4GlcNAcβ1-4GlcNAcβ-Sp24
58	Fucα1-2Galβ1-3GalNAcβ1-3Galα-Sp9
59	Fucα1-2Galβ1-3GalNAcβ1-3Galα1-4Galβ1-4Glcβ-Sp9
60	Fucα1-2Galβ1-3(Fucα1-4)GlcNAcβ-Sp8
61	Fucα1-2Galβ1-3GalNAca-Sp8
62	Fucα1-2Galβ1-3GalNAca-Sp14
63	Fucα1-2Galβ1-3GalNAcβ1-4(Neu5Aca2-3)Galβ1-4Glcβ-Sp0
64	Fucα1-2Galβ1-3GalNAcβ1-4(Neu5Aca2-3)Galβ1-4Glcβ-Sp9
65	Fucα1-2Galβ1-3GlcNAcβ1-3Galβ1-4Glcβ-Sp8
66	Fucα1-2Galβ1-3GlcNAcβ1-3Galβ1-4Glcβ-Sp10
67	Fucα1-2Galβ1-3GlcNAcβ-Sp0
68	Fucα1-2Galβ1-3GlcNAcβ-Sp8
69	Fucα1-2Galβ1-4(Fucα1-3)GlcNAcβ1-3Galβ1-4(Fucα1-3)GlcNAcβ-Sp0
70	Fucα1-2Galβ1-4(Fucα1-3)GlcNAcβ1-3Galβ1-4(Fucα1-3)GlcNAcβ1-3Galβ1-4(Fucα1-3)GlcNAcβ-Sp0
71	Fucα1-2Galβ1-4(Fucα1-3)GlcNAcβ-Sp0
72	Fucα1-2Galβ1-4(Fucα1-3)GlcNAcβ-Sp8
73	Fucα1-2Galβ1-4GlcNAcβ1-3Galβ1-4GlcNAcβ-Sp0
74	Fucα1-2Galβ1-4GlcNAcβ1-3Galβ1-4GlcNAcβ1-3Galβ1-4GlcNAcβ-Sp0
75	Fuca1-2Galb1-4GlcNAcb-Sp0
76	Fuca1-2Galb1-4GlcNAcb-Sp8
77	Fuca1-2Galb1-4Glcb-Sp0
78	Fuca1-2Galb-Sp8
79	Fuca1-3GlcNAcb-Sp8
80	Fuca1-4GlcNAcb-Sp8
81	Fucb1-3GlcNAcb-Sp8
82	GalNAca1-3(Fuca1-2)Galb1-3GlcNAcb-Sp0
83	GalNAca1-3(Fuca1-2)Galb1-4(Fuca1-3)GlcNAcb-Sp0
84	(3S)Galb1-4(Fuca1-3)Glcb-Sp0
85	GalNAca1-3(Fuca1-2)Galb1-4GlcNAcb-Sp0
86	GalNAca1-3(Fuca1-2)Galb1-4GlcNAcb-Sp8
87	GalNAca1-3(Fuca1-2)Galb1-4Glcb-Sp0

88	GlcNAcb1-3Galb1-3GalNAca-Sp8
89	GalNAca1-3(Fuca1-2)Galb-Sp8
90	GalNAca1-3(Fuca1-2)Galb-Sp18
91	GalNAca1-3GalNAcb-Sp8
92	GalNAca1-3Galb-Sp8
93	GalNAca1-4(Fuca1-2)Galb1-4GlcNAcb-Sp8
94	GalNAcb1-3GalNAca-Sp8
95	GalNAcb1-3(Fuca1-2)Galb-Sp8
96	GalNAcb1-3Gala1-4Galb1-4GlcNAcb-Sp0
97	GalNAcb1-4(Fuca1-3)GlcNAcb-Sp0
98	GalNAcb1-4GlcNAcb-Sp0
99	GalNAcb1-4GlcNAcb-Sp8
100	Gala1-2Galb-Sp8
101	Gala1-3(Fuca1-2)Galb1-3GlcNAcb-Sp0
102	Gala1-3(Fuca1-2)Galb1-3GlcNAcb-Sp8
103	Gala1-3(Fuca1-2)Galb1-4(Fuca1-3)GlcNAcb-Sp0
104	Gala1-3(Fuca1-2)Galb1-4(Fuca1-3)GlcNAcb-Sp8
105	Gala1-3(Fuca1-2)Galb1-4GlcNAc-Sp0
106	Gala1-3(Fuca1-2)Galb1-4Glcb-Sp0
107	Gala1-3(Fuca1-2)Galb-Sp8
108	Gala1-3(Fuca1-2)Galb-Sp18
109	Gala1-4(Gala1-3)Galb1-4GlcNAcb-Sp8
110	Gala1-3GalNAca-Sp8
111	Gala1-3GalNAca-Sp16
112	Gala1-3GalNAcb-Sp8
113	Gala1-3Galb1-4(Fuca1-3)GlcNAcb-Sp8
114	Gala1-3Galb1-3GlcNAcb-Sp0
115	Gala1-3Galb1-4GlcNAcb-Sp8
116	Gala1-3Galb1-4Glcb-Sp0
117	Gala1-3Galb1-4Glc-Sp10
118	Gala1-3Galb-Sp8
119	Gala1-4(Fuca1-2)Galb1-4GlcNAcb-Sp8
120	Gala1-4Galb1-4GlcNAcb-Sp0
121	Gala1-4Galb1-4GlcNAcb-Sp8
122	Gala1-4Galb1-4Glcb-Sp0
123	Gala1-4GlcNAcb-Sp8
124	Gala1-6Glcb-Sp8
125	Galb1-2Galb-Sp8
126	Galb1-3(Fuca1-4)GlcNAcb1-3Galb1-4(Fuca1-3)GlcNAcb-Sp0
127	Galb1-3GlcNAcb1-3Galb1-4(Fuca1-3)GlcNAcb-Sp0
128	Galb1-3(Fuca1-4)GlcNAc-Sp0
129	Galb1-3(Fuca1-4)GlcNAc-Sp8
130	Fuca1-4(Galb1-3)GlcNAcb-Sp8
131	Galb1-4GlcNAcb1-6GalNAca-Sp8
132	Galb1-4GlcNAcb1-6GalNAc-Sp14
133	GlcNAcb1-6(Galb1-3)GalNAca-Sp8

134	GlcNAcb1-6(Galb1-3)GalNAca-Sp14
135	Neu5Aca2-6(Galb1-3)GalNAca-Sp8
136	Neu5Aca2-6(Galb1-3)GalNAca-Sp14
137	Neu5Acb2-6(Galb1-3)GalNAca-Sp8
138	Neu5Aca2-6(Galb1-3)GlcNAcb1-4Galb1-4Glcb-Sp10
139	Galb1-3GalNAca-Sp8
140	Galb1-3GalNAca-Sp14
141	Galb1-3GalNAca-Sp16
142	Galb1-3GalNAcb-Sp8
143	Galb1-3GalNAcb1-3Gala1-4Galb1-4Glcb-Sp0
144	Galb1-3GalNAcb1-4(Neu5Aca2-3)Galb1-4Glcb-Sp0
145	Galb1-3GalNAcb1-4Galb1-4Glcb-Sp8
146	Galb1-3Galb-Sp8
147	Galb1-3GlcNAcb1-3Galb1-4GlcNAcb-Sp0
148	Galb1-3GlcNAcb1-3Galb1-4Glcb-Sp10
149	Galb1-3GlcNAcb-Sp0
150	Galb1-3GlcNAcb-Sp8
151	Galb1-4(Fuca1-3)GlcNAcb-Sp0
152	Galb1-4(Fuca1-3)GlcNAcb-Sp8
153	Galb1-4(Fuca1-3)GlcNAcb1-3Galb1-4(Fuca1-3)GlcNAcb-Sp0
154	Galb1-4(Fuca1-3)GlcNAcb1-3Galb1-4(Fuca1-3)GlcNAcb1-3Galb1-4(Fuca1-3)GlcNAcb-Sp0
155	Galb1-4(6S)Glcb-Sp0
156	Galb1-4(6S)Glcb-Sp8
157	Galb1-4GalNAca1-3(Fuca1-2)Galb1-4GlcNAcb-Sp8
158	Galb1-4GalNAcb1-3(Fuca1-2)Galb1-4GlcNAcb-Sp8
159	Galb1-4GlcNAcb1-3GalNAca-Sp8
160	Galb1-4GlcNAcb1-3GalNAc-Sp14
161	Galb1-4GlcNAcb1-3Galb1-4(Fuca1-3)GlcNAcb1-3Galb1-4(Fuca1-3)GlcNAcb-Sp0
162	Galb1-4GlcNAcb1-3Galb1-4GlcNAcb1-3Galb1-4GlcNAcb-Sp0
163	Galb1-4GlcNAcb1-3Galb1-4GlcNAcb-Sp0
164	Galb1-4GlcNAcb1-3Galb1-4Glcb-Sp0
165	Galb1-4GlcNAcb1-3Galb1-4Glcb-Sp8
166	Galb1-4GlcNAcb1-6(Galb1-3)GalNAca-Sp8
167	Galb1-4GlcNAcb1-6(Galb1-3)GalNAc-Sp14
168	Galb1-4GlcNAcb-Sp0
169	Galb1-4GlcNAcb-Sp8
170	Galb1-4GlcNAcb-Sp23
171	Galb1-4Glcb-Sp0
172	Galb1-4Glcb-Sp8
173	GlcNAca1-3Galb1-4GlcNAcb-Sp8
174	GlcNAca1-6Galb1-4GlcNAcb-Sp8
175	GlcNAcb1-2Galb1-3GalNAca-Sp8
176	GlcNAcb1-6(GlcNAcb1-3)GalNAca-Sp8
177	GlcNAcb1-6(GlcNAcb1-3)GalNAca-Sp14
178	GlcNAcb1-6(GlcNAcb1-3)Galb1-4GlcNAcb-Sp8
179	GlcNAcb1-3GalNAca-Sp8

180	GlcNAcb1-3GalNAca-Sp14
181	GlcNAcb1-3Galb-Sp8
182	GlcNAcb1-3Galb1-4GlcNAcb-Sp0
183	GlcNAcb1-3Galb1-4GlcNAcb-Sp8
184	GlcNAcb1-3Galb1-4GlcNAcb1-3Galb1-4GlcNAcb-Sp0
185	GlcNAcb1-3Galb1-4Glcb-Sp0
186	GlcNAcb1-4-MDPLys
187	GlcNAcb1-6(GlcNAcb1-4)GalNAca-Sp8
188	GlcNAcb1-4Galb1-4GlcNAcb-Sp8
189	GlcNAcb1-4GlcNAcb1-4GlcNAcb1-4GlcNAcb1-4GlcNAcb1-4GlcNAcb1-Sp8
190	GlcNAcb1-4GlcNAcb1-4GlcNAcb1-4GlcNAcb1-4GlcNAcb1-Sp8
191	GlcNAcb1-4GlcNAcb1-4GlcNAcb-Sp8
192	GlcNAcb1-6GalNAca-Sp8
193	GlcNAcb1-6GalNAca-Sp14
194	GlcNAcb1-6Galb1-4GlcNAcb-Sp8
195	Glca1-4Glcb-Sp8
196	Glca1-4Glca-Sp8
197	Glca1-6Glca1-6Glcb-Sp8
198	Glcb1-4Glcb-Sp8
199	Glcb1-6Glcb-Sp8
200	G-ol-Sp8
201	GlcAa-Sp8
202	GlcAb-Sp8
203	GlcAb1-3Galb-Sp8
204	GlcAb1-6Galb-Sp8
205	KDNa2-3Galb1-3GlcNAcb-Sp0
206	KDNa2-3Galb1-4GlcNAcb-Sp0
207	Mana1-2Mana1-2Mana1-3Mana-Sp9
208	Mana1-2Mana1-6(Mana1-2Mana1-3)Mana-Sp9
209	Mana1-2Mana1-3Mana-Sp9
210	Mana1-6(Mana1-2Mana1-3)Mana1-6(Mana1-2Mana1-3)Manb1-4GlcNAcb1-4GlcNAcb-Sp12
211	Mana1-2Mana1-6(Mana1-3)Mana1-6(Mana1-2Mana1-2Mana1-3)Manb1-4GlcNAcb1-4GlcNAcb-Sp12
212	Mana1-2Mana1-6(Mana1-2Mana1-3)Mana1-6(Mana1-2Mana1-2Mana1-3)Manb1-4GlcNAcb1-4GlcNAcb-Sp12
213	Mana1-6(Mana1-3)Mana-Sp9
214	Mana1-2Mana1-2Mana1-6(Mana1-3)Mana-Sp9
215	Mana1-6(Mana1-3)Mana1-6(Mana1-2Mana1-3)Manb1-4GlcNAcb1-4GlcNAcb-Sp12
216	Mana1-6(Mana1-3)Mana1-6(Mana1-3)Manb1-4GlcNAcb1-4GlcNAcb-Sp12
217	Manb1-4GlcNAcb-Sp0
218	Neu5Aca2-3Galb1-4GlcNAcb1-3Galb1-4(Fuca1-3)GlcNAcb-Sp0
219	(3S)Galb1-4(Fuca1-3)(6S)GlcNAcb-Sp8
220	Fuca1-2(6S)Galb1-4GlcNAcb-Sp0
221	Fuca1-2Galb1-4(6S)GlcNAcb-Sp8
222	Fuca1-2(6S)Galb1-4(6S)Glcb-Sp0
223	Neu5Aca2-3Galb1-3GalNAca-Sp8
224	Neu5Aca2-3Galb1-3GalNAca-Sp14

225	GalNAcb1-4(Neu5Aca2-8Neu5Aca2-8Neu5Aca2-8Neu5Aca2-3)Galb1-4Glcb-Sp0
226	GalNAcb1-4(Neu5Aca2-8Neu5Aca2-8Neu5Aca2-3)Galb1-4Glcb-Sp0
227	Neu5Aca2-8Neu5Aca2-8Neu5Aca2-3Galb1-4Glcb-Sp0
228	GalNAcb1-4(Neu5Aca2-8Neu5Aca2-3)Galb1-4Glcb-Sp0
229	Neu5Aca2-8Neu5Aca2-8Neu5Aca-Sp8
230	Neu5Aca2-3(6S)Galb1-4(Fuca1-3)GlcNAcb-Sp8
231	GalNAcb1-4(Neu5Aca2-3)Galb1-4GlcNAcb-Sp0
232	GalNAcb1-4(Neu5Aca2-3)Galb1-4GlcNAcb-Sp8
233	GalNAcb1-4(Neu5Aca2-3)Galb1-4Glcb-Sp0
234	Neu5Aca2-3Galb1-3GalNAcb1-4(Neu5Aca2-3)Galb1-4Glcb-Sp0
235	Neu5Aca2-6(Neu5Aca2-3)GalNAca-Sp8
236	Neu5Aca2-3GalNAca-Sp8
237	Neu5Aca2-3GalNAcb1-4GlcNAcb-Sp0
238	Neu5Aca2-3Galb1-3(6S)GlcNAc-Sp8
239	Neu5Aca2-3Galb1-3(Fuca1-4)GlcNAcb-Sp8
240	Neu5Aca2-3Galb1-3(Fuca1-4)GlcNAcb1-3Galb1-4(Fuca1-3)GlcNAcb-Sp0
241	Neu5Aca2-3Galb1-4(Neu5Aca2-3Galb1-3)GlcNAcb-Sp8
242	Neu5Aca2-3Galb1-3(6S)GalNAca-Sp8
243	Neu5Aca2-6(Neu5Aca2-3Galb1-3)GalNAca-Sp8
244	Neu5Aca2-6(Neu5Aca2-3Galb1-3)GalNAca-Sp14
245	Neu5Aca2-3Galb-Sp8
246	Neu5Aca2-3Galb1-3GalNAcb1-3Gala1-4Galb1-4Glcb-Sp0
247	Neu5Aca2-3Galb1-3GlcNAcb1-3Galb1-4GlcNAcb-Sp0
248	Fuca1-2(6S)Galb1-4Glcb-Sp0
249	Neu5Aca2-3Galb1-3GlcNAcb-Sp0
250	Neu5Aca2-3Galb1-3GlcNAcb-Sp8
251	Neu5Aca2-3Galb1-4(6S)GlcNAcb-Sp8
252	Neu5Aca2-3Galb1-4(Fuca1-3)(6S)GlcNAcb-Sp8
253	Neu5Aca2-3Galb1-4(Fuca1-3)GlcNAcb1-3Galb1-4(Fuca1-3)GlcNAcb1-3Galb1-4(Fuca1-3)GlcNAcb-Sp0
254	Neu5Aca2-3Galb1-4(Fuca1-3)GlcNAcb-Sp0
255	Neu5Aca2-3Galb1-4(Fuca1-3)GlcNAcb-Sp8
256	Neu5Aca2-3Galb1-4(Fuca1-3)GlcNAcb1-3Galb-Sp8
257	Neu5Aca2-3Galb1-4(Fuca1-3)GlcNAcb1-3Galb1-4GlcNAcb-Sp8
258	Neu5Aca2-3Galb1-4GlcNAcb1-3Galb1-4GlcNAcb1-3Galb1-4GlcNAcb-Sp0
259	Neu5Aca2-3Galb1-4GlcNAcb-Sp0
260	Neu5Aca2-3Galb1-4GlcNAcb-Sp8
261	Neu5Aca2-3Galb1-4GlcNAcb1-3Galb1-4GlcNAcb-Sp0
262	Fuca1-2Galb1-4(6S)Glcb-Sp0
263	Neu5Aca2-3Galb1-4Glcb-Sp0
264	Neu5Aca2-3Galb1-4Glcb-Sp8
265	Neu5Aca2-6GalNAca-Sp8
266	Neu5Aca2-6GalNAcb1-4GlcNAcb-Sp0
267	Neu5Aca2-6Galb1-4(6S)GlcNAcb-Sp8
268	Neu5Aca2-6Galb1-4GlcNAcb-Sp0
269	Neu5Aca2-6Galb1-4GlcNAcb-Sp8
270	Neu5Aca2-6Galb1-4GlcNAcb1-3Galb1-4(Fuca1-3)GlcNAcb1-3Galb1-4(Fuca1-3)GlcNAcb-Sp0

271	Neu5Aca2-6Galb1-4GlcNAcb1-3Galb1-4GlcNAcb-Sp0
272	Neu5Aca2-6Galb1-4Glcb-Sp0
273	Neu5Aca2-6Galb1-4Glcb-Sp8
274	Neu5Aca2-6Galb-Sp8
275	Neu5Aca2-8Neu5Aca-Sp8
276	Neu5Aca2-8Neu5Aca2-3Galb1-4Glcb-Sp0
277	Galb1-3(Fuca1-4)GlcNAcb1-3Galb1-3(Fuca1-4)GlcNAcb-Sp0
278	Neu5Acb2-6GalNAca-Sp8
279	Neu5Acb2-6Galb1-4GlcNAcb-Sp8
280	Neu5Gca2-3Galb1-3(Fuca1-4)GlcNAcb-Sp0
281	Neu5Gca2-3Galb1-3GlcNAcb-Sp0
282	Neu5Gca2-3Galb1-4(Fuca1-3)GlcNAcb-Sp0
283	Neu5Gca2-3Galb1-4GlcNAcb-Sp0
284	Neu5Gca2-3Galb1-4Glcb-Sp0
285	Neu5Gca2-6GalNAca-Sp0
286	Neu5Gca2-6Galb1-4GlcNAcb-Sp0
287	Neu5Gca-Sp8
288	Neu5Aca2-3Galb1-4GlcNAcb1-6(Galb1-3)GalNAca-Sp14
289	Galb1-3GlcNAcb1-3Galb1-3GlcNAcb-Sp0
290	Galb1-4(Fuca1-3)(6S)GlcNAcb-Sp0
291	Galb1-4(Fuca1-3)(6S)Glcb-Sp0
292	Galb1-4(Fuca1-3)GlcNAcb1-3Galb1-3(Fuca1-4)GlcNAcb-Sp0
293	Galb1-4GlcNAcb1-3Galb1-3GlcNAcb-Sp0
294	Neu5Aca2-3Galb1-3GlcNAcb1-3Galb1-3GlcNAcb-Sp0
295	Neu5Aca2-3Galb1-4GlcNAcb1-3Galb1-3GlcNAcb-Sp0
296	4S(3S)Galb1-4GlcNAcb-Sp0
297	(6S)Galb1-4(6S)GlcNAcb-Sp0
298	(6P)Glcb-Sp10
299	Neu5Aca2-3Galb1-4(Fuca1-3)GlcNAcb1-6(Galb1-3)GalNAca-Sp14
300	Galb1-3Galb1-4GlcNAcb-Sp8
301	Neu5Aca2-6Galb1-4GlcNAcb1-2Mana1-6(Galb1-4GlcNAcb1-2Mana1-3)Manb1-4GlcNAcb1-4GlcNAcb-Sp12
302	Galb1-4GlcNAcb1-6(Galb1-4GlcNAcb1-3)Galb1-4GlcNAc-Sp0
303	GlcNAcb1-6(Galb1-4GlcNAcb1-3)Galb1-4GlcNAc-Sp0
304	Galb1-4GlcNAca1-6Galb1-4GlcNAcb-Sp0
305	Galb1-4GlcNAcb1-6Galb1-4GlcNAcb-Sp0
306	GalNAcb1-3Galb-Sp8
307	GlcAb1-3GlcNAcb-Sp8
308	Neu5Aca2-6Galb1-4GlcNAcb1-2Mana1-6(GlcNAcb1-2Mana1-3)Manb1-4GlcNAcb1-4GlcNAcb-Sp12
309	GlcNAcb1-3Man-Sp10
310	GlcNAcb1-4GlcNAcb-Sp10
311	GlcNAcb1-4GlcNAcb-Sp12
312	MurNAcb1-4GlcNAcb-Sp10
313	Mana1-6Manb-Sp10
314	Mana1-6(Mana1-3)Mana1-6(Mana1-3)Manb-Sp10
315	Mana1-2Mana1-6(Mana1-3)Mana1-6(Mana1-2Mana1-2Mana1-3)Mana-Sp9
316	Mana1-2Mana1-6(Mana1-2Mana1-3)Mana1-6(Mana1-2Mana1-2Mana1-3)Mana-Sp9

317	Neu5Aca2-3Galb1-4GlcNAcb1-6(Neu5Aca2-3Galb1-3)GalNAca-Sp14
318	Neu5Aca2-6Galb1-4GlcNAcb1-2Mana1-6(Neu5Aca2-3Galb1-4GlcNAcb1-2Mana1-3)Manb1-4GlcNAcb1-4GlcNAcb-Sp12
319	Galb1-4GlcNAcb1-2Mana1-6(Neu5Aca2-6Galb1-4GlcNAcb1-2Mana1-3)Manb1-4GlcNAcb1-4GlcNAcb-Sp12
320	Neu5Aca2-8Neu5Acb-Sp17
321	Neu5Aca2-8Neu5Aca2-8Neu5Acb-Sp8
322	Neu5Gcb2-6Galb1-4GlcNAc-Sp8
323	Galb1-3GlcNAcb1-2Mana1-6(Galb1-3GlcNAcb1-2Mana1-3)Manb1-4GlcNAcb1-4GlcNAcb-Sp19
324	Neu5Aca2-3Galb1-4GlcNAcb1-2Mana1-6(Neu5Aca2-3Galb1-4GlcNAcb1-2Mana1-3)Manb1-4GlcNAcb1-4GlcNAcb-Sp12
325	Neu5Aca2-3Galb1-4GlcNAcb1-2Mana1-6(Neu5Aca2-6Galb1-4GlcNAcb1-2Mana1-3)Manb1-4GlcNAcb1-4GlcNAcb-Sp12
326	Galb1-4(Fuca1-3)GlcNAcb1-2Mana1-6(Galb1-4(Fuca1-3)GlcNAcb1-2Mana1-3)Manb1-4GlcNAcb1-4GlcNAcb-Sp20
327	Neu5,9Ac2a2-3Galb1-4GlcNAcb-Sp0
328	Neu5,9Ac2a2-3Galb1-3GlcNAcb-Sp0
329	Neu5Aca2-6Galb1-4GlcNAcb1-3Galb1-3GlcNAcb-Sp0
330	Neu5Aca2-3Galb1-3(Fuca1-4)GlcNAcb1-3Galb1-3(Fuca1-4)GlcNAcb-Sp0
331	Neu5Aca2-6Galb1-4GlcNAcb1-3Galb1-4GlcNAcb1-3Galb1-4GlcNAcb-Sp0
332	Gala1-4Galb1-4GlcNAcb1-3Galb1-4Glcb-Sp0
333	GalNAcb1-3Gala1-4Galb1-4GlcNAcb1-3Galb1-4Glcb-Sp0
334	GalNAca1-3(Fuca1-2)Galb1-4GlcNAcb1-3Galb1-4GlcNAcb-Sp0
335	GalNAca1-3(Fuca1-2)Galb1-4GlcNAcb1-3Galb1-4GlcNAcb1-3Galb1-4GlcNAcb-Sp0
336	Neu5Aca2-3Galb1-4(Fuca1-3)GlcNAcb1-6(Neu5Aca2-3Galb1-3)GalNAc-Sp14
337	GlcNAca1-4Galb1-4GlcNAcb1-3Galb1-4GlcNAcb1-3Galb1-4GlcNAcb-Sp0
338	GlcNAca1-4Galb1-4GlcNAcb-Sp0
339	GlcNAca1-4Galb1-3GlcNAcb-Sp0
340	GlcNAca1-4Galb1-4GlcNAcb1-3Galb1-4Glcb-Sp0
341	GlcNAca1-4Galb1-4GlcNAcb1-3Galb1-4(Fuca1-3)GlcNAcb1-3Galb1-4(Fuca1-3)GlcNAcb-Sp0
342	GlcNAca1-4Galb1-4GlcNAcb1-3Galb1-4GlcNAcb-Sp0
343	GlcNAca1-4Galb1-3GalNAc-Sp14
344	Neu5Aca2-6Galb1-4GlcNAcb1-2Mana1-6(Mana1-3)Manb1-4GlcNAcb1-4GlcNAc-Sp12
345	Mana1-6(Neu5Aca2-6Galb1-4GlcNAcb1-2Mana1-3)Manb1-4GlcNAcb1-4GlcNAc-Sp12
346	Neu5Aca2-6Galb1-4GlcNAcb1-2Mana1-6Manb1-4GlcNAcb1-4GlcNAc-Sp12
347	Neu5Aca2-6Galb1-4GlcNAcb1-2Mana1-3Manb1-4GlcNAcb1-4GlcNAc-Sp12
348	Galb1-4GlcNAcb1-2Mana1-3Manb1-4GlcNAcb1-4GlcNAc-Sp12
349	Galb1-4GlcNAcb1-2Mana1-6Manb1-4GlcNAcb1-4GlcNAc-Sp12
350	Mana1-6(Galb1-4GlcNAcb1-2Mana1-3)Manb1-4GlcNAcb1-4GlcNAcb-Sp12
351	GlcNAcb1-2Mana1-6(GlcNAcb1-2Mana1-3)Manb1-4GlcNAcb1-4(Fuca1-6)GlcNAcb-Sp22
352	Galb1-4GlcNAcb1-2Mana1-6(Galb1-4GlcNAcb1-2Mana1-3)Manb1-4GlcNAcb1-4(Fuca1-6)GlcNAcb-Sp22
353	Galb1-3GlcNAcb1-2Mana1-6(Galb1-3GlcNAcb1-2Mana1-3)Manb1-4GlcNAcb1-4(Fuca1-6)GlcNAcb-Sp22
354	(6S)GlcNAcb1-3Galb1-4GlcNAcb-Sp0
355	KDNa2-3Galb1-4(Fuca1-3)GlcNAc-Sp0
356	KDNa2-6Galb1-4GlcNAc-Sp0
357	KDNa2-3Galb1-4Glc-Sp0
358	KDNa2-3Galb1-3GalNAca-Sp14
359	Fuca1-2Galb1-3GlcNAcb1-2Mana1-6(Fuca1-2Galb1-3GlcNAcb1-2Mana1-3)Manb1-4GlcNAcb1-4GlcNAcb-Sp20
360	Fuca1-2Galb1-4GlcNAcb1-2Mana1-6(Fuca1-2Galb1-4GlcNAcb1-2Mana1-3)Manb1-4GlcNAcb1-4GlcNAcb-Sp20
361	Fuca1-2Galb1-4(Fuca1-3)GlcNAcb1-2Mana1-6(Fuca1-2Galb1-4(Fuca1-3)GlcNAcb1-2Mana1-3)Manb1-4GlcNAcb1-4GlcNAb-Sp20
362	Gala1-3Galb1-4GlcNAcb1-2Mana1-6(Gala1-3Galb1-4GlcNAcb1-2Mana1-3)Manb1-4GlcNAcb1-4GlcNAcb-Sp20

363	Galb1-4GlcNAcb1-2Mana1-6(Mana1-3)Manb1-4GlcNAcb1-4GlcNAcb-Sp12
364	Fuca1-4(Galb1-3)GlcNAcb1-2Mana1-6(Fuca1-4(Galb1-3)GlcNAcb1-2Mana1-3)Manb1-4GlcNAcb1-4(Fuca1-6)GlcNAcb-Sp22
365	Neu5Aca2-6GlcNAcb1-4GlcNAc-Sp21
366	Neu5Aca2-6GlcNAcb1-4GlcNAcb1-4GlcNAc-Sp21
367	Galb1-4(Fuca1-3)GlcNAcb1-6(Fuca1-2Galb1-4GlcNAcb1-3)Galb1-4Glc-Sp21
368	Galb1-4GlcNAcb1-2Mana1-6(Galb1-4GlcNAcb1-4(Galb1-4GlcNAcb1-2)Mana1-3)Manb1-4GlcNAcb1-4GlcNAc-Sp21
369	GalNAca1-3(Fuca1-2)Galb1-4GlcNAcb1-2Mana1-6(GalNAca1-3(Fuca1-2)Galb1-4GlcNAcb1-2Mana1-3)Manb1-4GlcNAcb1-4GlcNAcb-Sp20
370	Gala1-3(Fuca1-2)Galb1-4GlcNAcb1-2Mana1-6(Gala1-3(Fuca1-2)Galb1-4GlcNAcb1-2Mana1-3)Manb1-4GlcNAcb1-4GlcNAcb-Sp20
371	Gala1-3Galb1-4(Fuca1-3)GlcNAcb1-2Mana1-6(Gala1-3Galb1-4(Fuca1-3)GlcNAcb1-2Mana1-3)Manb1-4GlcNAcb1-4GlcNAcb-Sp20
372	GalNAca1-3(Fuca1-2)Galb1-3GlcNAcb1-2Mana1-6(GalNAca1-3(Fuca1-2)Galb1-3GlcNAcb1-2Mana1-3)Manb1-4GlcNAcb1-4GlcNAcb-Sp20
373	Gala1-3(Fuca1-2)Galb1-3GlcNAcb1-2Mana1-6(Gala1-3(Fuca1-2)Galb1-3GlcNAcb1-2Mana1-3)Manb1-4GlcNAcb1-4GlcNAcb-Sp20
374	Fuca1-4(Fuca1-2Galb1-3)GlcNAcb1-2Mana1-3(Fuca1-4(Fuca1-2Galb1-3)GlcNAcb1-2Mana1-3)Manb1-4GlcNAcb1-4GlcNAcb-Sp19
375	Neu5Aca2-3Galb1-4GlcNAcb1-3GalNAc-Sp14
376	Neu5Aca2-6Galb1-4GlcNAcb1-3GalNAc-Sp14
377	Neu5Aca2-3Galb1-4(Fuca1-3)GlcNAcb1-3GalNAca-Sp14
378	GalNAcb1-4GlcNAcb1-2Mana1-6(GalNAcb1-4GlcNAcb1-2Mana1-3)Manb1-4GlcNAcb1-4GlcNAc-Sp12
379	Galb1-3GalNAca1-3(Fuca1-2)Galb1-4Glc-Sp0
380	Galb1-3GalNAca1-3(Fuca1-2)Galb1-4GlcNAc-Sp0
381	Galb1-3GlcNAcb1-3Galb1-4GlcNAcb1-6(Galb1-3GlcNAcb1-3)Galb1-4Glcb-Sp0
382	Galb1-4(Fuca1-3)GlcNAcb1-6(Galb1-3GlcNAcb1-3)Galb1-4Glc-Sp21
383	Galb1-4GlcNAcb1-6(Fuca1-4(Fuca1-2Galb1-3)GlcNAcb1-3)Galb1-4Glc-Sp21
384	Galb1-4(Fuca1-3)GlcNAcb1-6(Fuca1-4(Fuca1-2Galb1-3)GlcNAcb1-3)Galb1-4Glc-Sp21
385	Galb1-3GlcNAcb1-3Galb1-4(Fuca1-3)GlcNAcb1-6(Galb1-3GlcNAcb1-3)Galb1-4Glc-Sp21
386	Galb1-4GlcNAcb1-6(Galb1-4GlcNAcb1-2)Mana1-6(Galb1-4GlcNAcb1-4(Galb1-4GlcNAcb1-2)Mana1-3)Manb1-4GlcNAcb1-4GlcNAcb-Sp21
387	GlcNAcb1-2Mana1-6(GlcNAcb1-4(GlcNAcb1-2)Mana1-3)Manb1-4GlcNAcb1-4GlcNAc-Sp21
388	Fuca1-2Galb1-3GalNAca1-3(Fuca1-2)Galb1-4Glcb-Sp0
389	Fuca1-2Galb1-3GalNAca1-3(Fuca1-2)Galb1-4GlcNAcb-Sp0
390	Galb1-3GlcNAcb1-3GalNAca-Sp14
391	GalNAcb1-4(Neu5Aca2-3)Galb1-4GlcNAcb1-3GalNAca-Sp14
392	GalNAca1-3(Fuca1-2)Galb1-3GalNAca1-3(Fuca1-2)Galb1-4GlcNAcb-Sp0
393	Gala1-3Galb1-3GlcNAcb1-2Mana1-6(Gala1-3Galb1-3GlcNAcb1-2Mana1-3)Manb1-4GlcNAcb1-4GlcNAc-Sp19
394	Gala1-3Galb1-3(Fuca1-4)GlcNAcb1-2Mana1-6(Gala1-3Galb1-3(Fuca1-4)GlcNAcb1-2Mana1-3)Manb1-4GlcNAcb1-4GlcNAc-Sp19
395	Neu5Aca2-3Galb1-3GlcNAcb1-2Mana1-6(Neu5Aca2-3Galb1-3GlcNAcb1-2Mana1-3)Manb1-4GlcNAcb1-4GlcNAc-Sp19
396	GlcNAcb1-2Mana1-6(Galb1-4GlcNAcb1-2Mana1-3)Manb1-4GlcNAcb1-4GlcNAc-Sp12
397	Galb1-4GlcNAcb1-2Mana1-6(GlcNAcb1-2Mana1-3)Manb1-4GlcNAcb1-4GlcNAc-Sp12
398	Neu5Aca2-3Galb1-3GlcNAcb1-3GalNAca-Sp14
399	Fuca1-2Galb1-4GlcNAcb1-3GalNAca-Sp14
400	Galb1-4(Fuca1-3)GlcNAcb1-3GalNAca-Sp14
401	GalNAca1-3GalNAcb1-3Gala1-4Galb1-4GlcNAcb-Sp0
402	Gala1-4Galb1-3GlcNAcb1-2Mana1-6(Gala1-4Galb1-3GlcNAcb1-2Mana1-3)Manb1-4GlcNAcb1-4GlcNAcb-Sp19
403	Gala1-4Galb1-4GlcNAcb1-2Mana1-6(Gala1-4Galb1-4GlcNAcb1-2Mana1-3)Manb1-4GlcNAcb1-4GlcNAcb-Sp24
404	Gala1-3Galb1-4GlcNAcb1-3GalNAca-Sp14
405	Galb1-3GlcNAcb1-6Galb1-4GlcNAcb-Sp0
406	Galb1-3GlcNAca1-6Galb1-4GlcNAcb-Sp0
407	GalNAcb1-3Gala1-6Galb1-4Glcb-Sp8

408	Gala1-3(Fuca1-2)Galb1-4(Fuca1-3)Glcb-Sp21
409	Galb1-4GlcNAcb1-6(Neu5Aca2-6Galb1-3GlcNAcb1-3)Galb1-4Glc-Sp21
410	Galb1-3GalNAcb1-4(Neu5Aca2-8Neu5Aca2-3)Galb1-4Glcb-Sp0
411	Neu5Aca2-3Galb1-3GalNAcb1-4(Neu5Aca2-8Neu5Aca2-3)Galb1-4Glcb-Sp0
412	Gala1-3(Fuca1-2)Galb1-4GlcNAcb1-3GalNAca-Sp14
413	GalNAca1-3(Fuca1-2)Galb1-4GlcNAcb1-3GalNAca-Sp14
414	GalNAca1-3GalNAcb1-3Gala1-4Galb1-4Glcb-Sp0
415	Fuca1-2Galb1-4(Fuca1-3)GlcNAcb1-3GalNAca-Sp14
416	Gala1-3(Fuca1-2)Galb1-4(Fuca1-3)GlcNAcb1-3GalNAc-Sp14
417	GalNAca1-3(Fuca1-2)Galb1-4(Fuca1-3)GlcNAcb1-3GalNAc-Sp14
418	Galb1-4(Fuca1-3)GlcNAcb1-2Mana1-6(Galb1-4(Fuca1-3)GlcNAcb1-2Mana1-3)Manb1-4GlcNAcb1-4(Fuca1-6)GlcNAcb-Sp22
419	Fuca1-2Galb1-4GlcNAcb1-2Mana1-6(Fuca1-2Galb1-4GlcNAcb1-2Mana1-3)Manb1-4GlcNAcb1-4(Fuca1-6)GlcNAcb-Sp22
420	GlcNAcb1-2(GlcNAcb1-6)Mana1-6(GlcNAcb1-2Mana1-3)Manb1-4GlcNAcb1-4GlcNAcb-Sp19
421	Fuca1-2Galb1-3GlcNAcb1-3GalNAc-Sp14
422	Gala1-3(Fuca1-2)Galb1-3GlcNAcb1-3GalNAc-Sp14
423	GalNAca1-3(Fuca1-2)Galb1-3GlcNAcb1-3GalNAc-Sp14
424	Gala1-3Galb1-3GlcNAcb1-3GalNAc-Sp14
425	Fuca1-2Galb1-3GlcNAcb1-2Mana1-6(Fuca1-2Galb1-3GlcNAcb1-2Mana1-3)Manb1-4GlcNAcb1-4(Fuca1-6)GlcNAcb-Sp22
426	Gala1-3(Fuca1-2)Galb1-4GlcNAcb1-2Mana1-6(Gala1-3(Fuca1-2)Galb1-4GlcNAcb1-2Mana1-3)Manb1-4GlcNAcb1-4(Fuca1-6)GlcNAcb-Sp22
427	Galb1-3GlcNAcb1-6(Galb1-3GlcNAcb1-2)Mana1-6(Galb1-3GlcNAcb1-2Mana1-3)Manb1-4GlcNAcb1-4GlcNAcb-Sp19
428	Galb1-4GlcNAcb1-6(Fuca1-2Galb1-3GlcNAcb1-3)Galb1-4Glc-Sp21
429	Fuca1-3GlcNAcb1-6(Galb1-4GlcNAcb1-3)Galb1-4Glc-Sp21
430	GlcNAcb1-2Mana1-6(GlcNAcb1-4)(GlcNAcb1-2Mana1-3)Manb1-4GlcNAcb1-4GlcNAc-Sp21
431	GlcNAcb1-2Mana1-6(GlcNAcb1-4)(GlcNAcb1-4(GlcNAcb1-2)Mana1-3)Manb1-4GlcNAcb1-4GlcNAc-Sp21
432	GlcNAcb1-6(GlcNAcb1-2)Mana1-6(GlcNAcb1-4)(GlcNAcb1-2Mana1-3)Manb1-4GlcNAcb1-4GlcNAc-Sp21
433	GlcNAcb1-6(GlcNAcb1-2)Mana1-6(GlcNAcb1-4)(GlcNAcb1-4(GlcNAcb1-2)Mana1-3)Manb1-4GlcNAcb1-4GlcNAc-Sp21
434	Galb1-4GlcNAcb1-2Mana1-6(GlcNAcb1-4)(Galb1-4GlcNAcb1-2Mana1-3)Manb1-4GlcNAcb1-4GlcNAc-Sp21
435	Galb1-4GlcNAcb1-2Mana1-6(GlcNAcb1-4)(Galb1-4GlcNAcb1-4(Galb1-4GlcNAcb1-2)Mana1-3)Manb1-4GlcNAcb1-4GlcNAc-Sp21
436	Galb1-4GlcNAcb1-6(Galb1-4GlcNAcb1-2)Mana1-6(GlcNAcb1-4)(Galb1-4GlcNAcb1-2Mana1-3)Manb1-4GlcNAcb1-4GlcNAc-Sp21
437	Galb1-4GlcNAcb1-6(Galb1-4GlcNAcb1-2)Mana1-6(GlcNAcb1-4)(Galb1-4GlcNAcb1-4(Galb1-4GlcNAcb1-2)Mana1-3)Manb1-4GlcNAcb1-4GlcNAc-Sp21
438	Galb1-4Galb-Sp10
439	Galb1-6Galb-Sp10
440	Neu5Aca2-3Galb1-4GlcNAcb1-3Galb-Sp8
441	GalNAcb1-6GalNAcb-Sp8
442	(6S)Galb1-3GlcNAcb-Sp0
443	(6S)Galb1-3(6S)GlcNAc-Sp0
444	Fuca1-2Galb1-4 GlcNAcb1-2Mana1-6(Fuca1-2Galb1-4GlcNAcb1-2(Fuca1-2Galb1-4GlcNAcb1-4)Mana1-3)Manb1-4GlcNAcb1-4GlcNAcb-Sp12
445	Fuca1-2Galb1-4(Fuca1-3)GlcNAcb1-2Mana1-6(Fuca1-2Galb1-4(Fuca1-3)GlcNAcb1-4(Fuca1-2Galb1-4(Fuca1-3)GlcNAcb1-2)Mana1-3)Manb1-4GlcNAcb1-4GlcNAcb-Sp12
446	Galb1-4(Fuca1-3)GlcNAcb1-6GalNAc-Sp14
447	Galb1-4GlcNAcb1-2Mana-Sp0
448	Fuca1-2Galb1-4GlcNAcb1-6(Fuca1-2Galb1-4GlcNAcb1-3)GalNAc-Sp14
449	Gala1-3(Fuca1-2)Galb1-4GlcNAcb1-6(Gala1-3(Fuca1-2)Galb1-4GlcNAcb1-3)GalNAc-Sp14
450	GalNAca1-3(Fuca1-2)Galb1-4GlcNAcb1-6(GalNAca1-3(Fuca1-2)Galb1-4GlcNAcb1-3)GalNAc-Sp14
451	Neu5Aca2-8Neu5Aca2-3Galb1-3GalNAcb1-4(Neu5Aca2-8Neu5Aca2-3)Galb1-4Glcb-Sp0
452	GalNAcb1-4Galb1-4Glcb-Sp0

453	GalNAca1-3(Fuca1-2)Galb1-4GlcNAcb1-2Mana1-6(GalNAca1-3(Fuca1-2)Galb1-4GlcNAcb1-2Mana1-3)Manb1-4GlcNAcb1-4(Fuca1-6)GlcNAcb-Sp22
454	Gala1-3(Fuca1-2)Galb1-3GlcNAcb1-2Mana1-6(Gala1-3(Fuca1-2)Galb1-3GlcNAcb1-2Mana1-3)Manb1-4GlcNAcb1-4(Fuca1-6)GlcNAcb-Sp22
455	Neu5Aca2-6Galb1-4GlcNAcb1-6(Fuca1-2Galb1-3GlcNAcb1-3)Galb1-4Glc-Sp21
456	GalNAca1-3(Fuca1-2)Galb1-3GlcNAcb1-2Mana1-6(GalNAca1-3(Fuca1-2)Galb1-3GlcNAcb1-2Mana1-3)Manb1-4GlcNAcb1-4(Fuca1-6)GlcNAcb-Sp22
457	Galb1-4GlcNAcb1-6(Galb1-4GlcNAcb1-2)Mana1-6(Galb1-4GlcNAcb1-2Mana1-3)Manb1-4GlcNAcb1-4GlcNAcb-Sp19
458	Neu5Aca2-3Galb1-4GlcNAcb1-2Mana1-6(GlcNAcb1-4)(Neu5Aca2-3Galb1-4GlcNAcb1-2Mana1-3)Manb1-4GlcNAcb1-4GlcNAcb-Sp21
459	Neu5Aca2-3Galb1-4GlcNAcb1-4Mana1-6(GlcNAcb1-4)(Neu5Aca2-3Galb1-4GlcNAcb1-4(Neu5Aca2-3Galb1-4GlcNAcb1-2)Mana1-3)Manb1-4GlcNAcb1-4GlcNAcb-Sp21
460	Neu5Aca2-3Galb1-4GlcNAcb1-6(Neu5Aca2-3Galb1-4GlcNAcb1-2)Mana1-6(GlcNAcb1-4)(Neu5Aca2-3Galb1-4GlcNAcb1-2Mana1-3)Manb1-4GlcNAcb1-4GlcNAcb-Sp21
461	Neu5Aca2-3Galb1-4GlcNAcb1-6(Neu5Aca2-3Galb1-4GlcNAcb1-2)Mana1-6(GlcNAcb1-4)(Neu5Aca2-3Galb1-4GlcNAcb1-4(Neu5Aca2-3Galb1-4GlcNAcb1-2)Mana1-3)Manb1-4GlcNAcb1-4GlcNAcb-Sp21
462	Neu5Aca2-6Galb1-4GlcNAcb1-2Mana1-6(GlcNAcb1-4)(Neu5Aca2-6Galb1-4GlcNAcb1-2Mana1-3)Manb1-4GlcNAcb1-4GlcNAcb-Sp21
463	Neu5Aca2-6Galb1-4GlcNAcb1-4Mana1-6(GlcNAcb1-4)(Neu5Aca2-6Galb1-4GlcNAcb1-4(Neu5Aca2-6Galb1-4GlcNAcb1-2)Mana1-3)Manb1-4GlcNAcb1-4GlcNAcb-Sp21
464	Neu5Aca2-6Galb1-4GlcNAcb1-6(Neu5Aca2-6Galb1-4GlcNAcb1-2)Mana1-6(GlcNAcb1-4)(Neu5Aca2-6Galb1-4GlcNAcb1-2Mana1-3)Manb1-4GlcNAcb1-4GlcNAcb-Sp21
465	Neu5Aca2-6Galb1-4GlcNAcb1-6(Neu5Aca2-6Galb1-4GlcNAcb1-2)Mana1-6(GlcNAcb1-4)(Neu5Aca2-6Galb1-4GlcNAcb1-4(Neu5Aca2-6Galb1-4GlcNAcb1-2)Mana1-3)Manb1-4GlcNAcb1-4GlcNAcb-Sp21
466	Gala1-3(Fuca1-2)Galb1-3GalNAca-Sp8
467	Gala1-3(Fuca1-2)Galb1-3GalNAcb-Sp8
468	Glca1-6Glca1-6Glca1-6Glcb-Sp10
469	Glca1-4Glca1-4Glca1-4Glcb-Sp10
470	Neu5Aca2-3Galb1-4GlcNAcb1-6(Neu5Aca2-3Galb1-4GlcNAcb1-3)GalNAca-Sp14
471	Fuca1-2Galb1-4(Fuca1-3)GlcNAcb1-2Mana1-6(Fuca1-2Galb1-4(Fuca1-3)GlcNAcb1-2Mana1-3)Manb1-4GlcNAcb1-4(Fuca1-6)GlcNAcb-Sp24
472	Fuca1-2Galb1-3(Fuca1-4)GlcNAcb1-2Mana1-6(Fuca1-2Galb1-3(Fuca1-4)GlcNAcb1-2Mana1-3)Manb1-4GlcNAcb1-4(Fuca1-6)GlcNAcb1-4(Fuca1-6)GlcNAcb-Sp19
473	Neu5Aca2-3Galb1-3GlcNAcb1-6(Neu5Aca2-3Galb1-3GlcNAcb1-2)Mana1-6(Neu5Aca2-3Galb1-3GlcNAcb1-2Mana1-3)Manb1-4GlcNAcb1-4GlcNAcb-Sp19
474	GlcNAcb1-6(GlcNAcb1-2)Mana1-6(GlcNAcb1-2Mana1-3)Manb1-4GlcNAcb1-4(Fuca1-6)GlcNAcb-Sp24
475	Galb1-3GlcNAcb1-2Mana1-6(GlcNAcb1-4)(Galb1-3GlcNAcb1-2Mana1-3)Manb1-4GlcNAcb1-4GlcNAcb-Sp21
476	Neu5Aca2-6Galb1-4GlcNAcb1-6(Galb1-3GlcNAcb1-3)Galb1-4Glcb-Sp21
477	Neu5Aca2-3Galb1-4GlcNAcb1-2Mana-Sp0
478	Neu5Aca2-3Galb1-4GlcNAcb1-6GalNAca-Sp14
479	Neu5Aca2-6Galb1-4GlcNAcb1-6GalNAca-Sp14
480	Neu5Aca2-6Galb1-4 GlcNAcb1-6(Neu5Aca2-6Galb1-4GlcNAcb1-3)GalNAca-Sp14
481	Neu5Aca2-6Galb1-4GlcNAcb1-2Mana1-6(Neu5Aca2-6Galb1-4GlcNAcb1-2Mana1-3)Manb1-4GlcNAcb1-4(Fuca1-6)GlcNAcb-Sp24
482	Neu5Aca2-3Galb1-4GlcNAcb1-2Mana1-6(Neu5Aca2-3Galb1-4GlcNAcb1-2Mana1-3)Manb1-4GlcNAcb1-4(Fuca1-6)GlcNAcb-Sp24
483	Mana1-6(Mana1-3)Manb1-4GlcNAcb1-4(Fuca1-6)GlcNAcb-Sp19
484	Galb1-4GlcNAcb1-6(Galb1-4GlcNAcb1-2)Mana1-6(Galb1-4GlcNAcb1-2Mana1-3)Manb1-4GlcNAcb1-4(Fuca1-6)GlcNAcb-Sp24
485	Neu5Aca2-3Galb1-3GlcNAcb1-2Mana1-6(GlcNAcb1-4)(Neu5Aca2-3Galb1-3GlcNAcb1-2Mana1-3)Manb1-4GlcNAcb1-4GlcNAc-Sp21
486	Neu5Aca2-6Galb1-4GlcNAcb1-6(Fuca1-2Galb1-4(Fuca1-3)GlcNAcb1-3)Galb1-4Glc-Sp21
487	Galb1-3GlcNAcb1-6GalNAca-Sp14
488	Gala1-3Galb1-3GlcNAcb1-6GalNAca-Sp14
489	Galb1-3(Fuca1-4)GlcNAcb1-6GalNAca-Sp14
490	Neu5Aca2-3Galb1-3GlcNAcb1-6GalNAca-Sp14
491	(3S)Galb1-3(Fuca1-4)GlcNAcb-Sp0
492	Galb1-4(Fuca1-3)GlcNAcb1-6(Neu5Aca2-6(Neu5Aca2-3Galb1-3)GlcNAcb1-3)Galb1-4Glc-Sp21
493	Fuca1-2Galb1-4GlcNAcb1-6GalNAca-Sp14
494	Gala1-3Galb1-4GlcNAcb1-6GalNAca-Sp14
495	Galb1-4(Fuca1-3)GlcNAcb1-2Mana-Sp0
496	Fuca1-2(6S)Galb1-3GlcNAcb-Sp0

497	Gala1-3(Fuca1-2)Galb1-4GlcNAcb1-6GalNAca-Sp14
498	Fuca1-2Galb1-4GlcNAcb1-2Mana-Sp0
499	Fuca1-2Galb1-3(6S)GlcNAcb-Sp0
500	Fuca1-2(6S)Galb1-3(6S)GlcNAcb-Sp0
501	Neu5Aca2-6GalNAcb1-4(6S)GlcNAcb-Sp8
502	GalNAcb1-4(Fuca1-3)(6S)GlcNAcb-Sp8
503	(3S)GalNAcb1-4(Fuca1-3)GlcNAcb-Sp8
504	Fuca1-2Galb1-3GlcNAcb1-6(Fuca1-2Galb1-3GlcNAcb1-3)GalNAca-Sp14
505	GalNAca1-3(Fuca1-2)Galb1-3GlcNAcb1-6GalNAca-Sp14
506	GlcNAcb1-6(GlcNAcb1-2)Mana1-6(GlcNAcb1-4)(GlcNAcb1-4(GlcNAcb1-2)Mana1-3)Manb1-4GlcNAcb1-4(Fuca1-6)GlcNAc-Sp21
507	Galb1-4GlcNAcb1-6(Galb1-4GlcNAcb1-2)Mana1-6(GlcNAcb1-4)Galb1-4GlcNAcb1-4(Gal b1-4GlcNAcb1-2)Mana1-3)Manb1-4GlcNAcb1-4(Fuca1-6)GlcNAc-Sp21
508	Galb1-3GlcNAca1-3Galb1-4GlcNAcb-Sp8
509	Galb1-3(6S)GlcNAcb-Sp8
510	(6S)(4S)GalNAcb1-4GlcNAc-Sp8
511	(6S)GalNAcb1-4GlcNAc-Sp8
512	(3S)GalNAcb1-4(3S)GlcNAc-Sp8
513	GalNAcb1-4(6S)GlcNAc-Sp8
514	(3S)GalNAcb1-4GlcNAc-Sp8
515	(4S)GalNAcb-Sp10
516	Galb1-4(6P)GlcNAcb-Sp0
517	(6P)Galb1-4GlcNAcb-SP0
518	GalNAca1-3(Fuca1-2)Galb1-4GlcNAcb1-6GalNAc-Sp14
519	Neu5Aca2-6Galb1-4GlcNAcb1-2Man-Sp0
520	Gala1-3Galb1-4GlcNAcb1-2Mana-Sp0
521	Gala1-3(Fuca1-2)Galb1-4GlcNAcb1-2Mana-Sp0
522	GalNAca1-3(Fuca1-2)Galb1-4 GlcNAcb1-2Mana-Sp0
523	Galb1-3GlcNAcb1-2Mana-Sp0
524	Gala1-3(Fuca1-2)Galb1-3GlcNAcb1-6GalNAc-Sp14
525	Neu5Aca2-3Galb1-3GlcNAcb1-2Mana-Sp0
526	Gala1-3Galb1-3GlcNAcb1-2Mana-Sp0
527	GalNAcb1-4GlcNAcb1-2Mana-Sp0
528	Neu5Aca2-3Galb1-3GalNAcb1-4Galb1-4Glcb-Sp0
529	GlcNAcb1-2 Mana1-6(GlcNAcb1-4)(GlcNAcb1-2Mana1-3)Manb1-4GlcNAcb1-4(Fuca1-6)GlcNAc-Sp21
530	Galb1-4GlcNAcb1-2 Mana1-6(GlcNAcb1-4)(Galb1-4GlcNAcb1-2Mana1-3)Manb1-4GlcNAcb1-4(Fuca1-6)GlcNAc-Sp21
531	Galb1-4GlcNAcb1-2 Mana1-6(Galb1-4GlcNAcb1-4)(Galb1-4GlcNAcb1-2Mana1-3)Manb1-4GlcNAcb1-4(Fuca1-6)GlcNAc-Sp21
532	Fuca1-4(Galb1-3)GlcNAcb1-2 Mana-Sp0
533	Neu5Aca2-3Galb1-4(Fuca1-3)GlcNAcb1-2Mana-Sp0
534	GlcNAcb1-3Galb1-4GlcNAcb1-6(GlcNAcb1-3)Galb1-4GlcNAc-Sp0
535	GalNAca1-3(Fuca1-2)Galb1-3GalNAcb1-3Gala1-4Galb1-4Glc-Sp21
536	Gala1-3(Fuca1-2)Galb1-3GalNAcb1-3Gala1-4Galb1-4Glc-Sp21
537	Galb1-3GalNAcb1-3Gal-Sp21
538	GlcNAcb1-3Galb1-4GlcNAcb1-2Mana1-6(GlcNAcb1-3Galb1-4GlcNAcb1-2Mana1-3)Manb1-4GlcNAcb1-4GlcNAcb-Sp12
539	GlcNAcb1-3Galb1-4GlcNAcb1-2Mana1-6(GlcNAcb1-3Galb1-4GlcNAcb1-2Mana1-3)Manb1-4GlcNAcb1-4GlcNAcb-Sp25
540	Galb1-4GlcNAcb1-3Galb1-4GlcNAcb1-2Mana1-6(Galb1-4GlcNAcb1-3Galb1-4GlcNAcb1-2Mana1-3)Manb1-4GlcNAcb1-4GlcNAcb-Sp12
541	Galb1-4GlcNAcb1-3Galb1-4GlcNAcb1-2Mana1-6(Galb1-4GlcNAcb1-3Galb1-4GlcNAcb1-2Mana1-3)Manb1-4GlcNAcb1-4GlcNAcb-Sp24

542	Neu5Gca2-3Galb1-4GlcNAcb1-3Galb1-4GlcNAcb1-2Mana1-6(Neu5Gca2-3Galb1-4GlcNAcb1-3Galb1-4GlcNAcb1-2Mana1-3)Manb1-4GlcNAcb1-4GlcNAcb-Sp24
543	Fuca1-2Galb1-4GlcNAcb1-3Galb1-4GlcNAcb1-2Mana1-6(Fuca1-2Galb1-4GlcNAcb1-3Galb1-4GlcNAcb1-2Mana1-3)Manb1-4GlcNAcb1-4GlcNAcb-Sp24
544	GlcNAcb1-3Galb1-4GlcNAcb1-3Galb1-4GlcNAcb1-2Mana1-6(GlcNAcb1-3Galb1-4GlcNAcb1-3Galb1-4GlcNAcb1-2Mana1-3)Manb1-4GlcNAcb1-4GlcNAcb-Sp12
545	GlcNAcb1-3Galb1-4GlcNAcb1-3Galb1-4GlcNAcb1-2Mana1-6(GlcNAcb1-3Galb1-4GlcNAcb1-3Galb1-4GlcNAcb1-2Mana1-3)Manb1-4GlcNAcb1-4GlcNAcb-Sp25
546	Galb1-4GlcNAcb1-3Galb1-4GlcNAcb1-3Galb1-4GlcNAcb1-2Mana1-6(Galb1-4GlcNAcb1-3Galb1-4GlcNAcb1-3Galb1-4GlcNAcb1-2Mana1-3)Manb1-4GlcNAcb1-4GlcNAcb-Sp12
547	Galb1-4GlcNAcb1-3Galb1-4GlcNAcb1-3Galb1-4GlcNAcb1-2Mana1-6(Galb1-4GlcNAcb1-3Galb1-4GlcNAcb1-3Galb1-4GlcNAcb1-2Mana1-3)Manb1-4GlcNAcb1-4GlcNAcb-Sp24
548	GlcNAcb1-3Galb1-4GlcNAcb1-3Galb1-4GlcNAcb1-3Galb1-4GlcNAcb1-2Mana1-6(GlcNAcb1-3Galb1-4GlcNAcb1-3Galb1-4GlcNAcb1-3Galb1-4GlcNAcb1-2Mana1-3)Manb1-4GlcNAcb1-4GlcNAcb-Sp25
549	Galb1-4GlcNAcb1-3Galb1-4GlcNAcb1-3Galb1-4GlcNAcb1-3Galb1-4GlcNAcb1-2Mana1-6(Galb1-4GlcNAcb1-3Galb1-4GlcNAcb1-3Galb1-4GlcNAcb1-3Galb1-4GlcNAcb1-2Mana1-3)Manb1-4GlcNAcb1-4GlcNAcb-Sp25
550	Galb1-3GlcNAcb1-3Galb1-4GlcNAcb1-2Mana1-6(Galb1-3GlcNAcb1-3Galb1-4GlcNAcb1-2Mana1-3)Manb1-4GlcNAcb1-4GlcNAc-Sp25
551	Neu5Gca2-8Neu5Gca2-3Galb1-4GlcNAc-Sp0
552	Neu5Aca2-8Neu5Gca2-3Galb1-4GlcNAc-Sp0
553	Neu5Gca2-8Neu5Aca2-3Galb1-4GlcNAc-Sp0
554	Neu5Gca2-8Neu5Gca2-3Galb1-4GlcNAcb1-3Galb1-4GlcNAc-Sp0
555	Neu5Gca2-8Neu5Gca2-6Galb1-4GlcNAc-Sp0
556	Neu5Aca2-8Neu5Aca2-3Galb1-4GlcNAc-Sp0
557	GlcNAcb1-3Galb1-4GlcNAcb1-6(GlcNAcb1-3Galb1-4GlcNAcb1-2)Mana1-6(GlcNAcb1-3Galb1-4GlcNAcb1-2Man a1-3)Manb1-4GlcNAcb1-4GlcNAc-Sp24
558	Galb1-4GlcNAcb1-3Galb1-4GlcNAcb1-6(Galb1-4GlcNAcb1-3Galb1-4GlcNAcb1-2)Mana1-6(Galb1-4GlcNAcb1-3Galb1-4GlcNAcb1-2Mana1-3)Mana1-4GlcNAcb1-4GlcNAc-Sp24
559	Gala1-3Galb1-4GlcNAcb1-2Mana1-6(Gala1-3Galb1-4GlcNAcb1-2Mana1-3)Manb1-4GlcNAcb1-4GlcNAc-Sp24
560	GlcNAcb1-3Galb1-4GlcNAcb1-6(GlcNAcb1-3Galb1-3)GalNAca-Sp14
561	GalNAcb1-3GlcNAcb-Sp0
562	GalNAcb1-4GlcNAcb1-3GalNAcb1-4GlcNAcb-Sp0
563	GlcNAcb1-3Galb1-4GlcNAcb1-3Galb1-4GlcNAcb1-3Galb1-4GlcNAcb1-3Galb1-4GlcNAcb1-2Mana1-6(GlcNAcb1-3Galb1-4GlcNAcb1-3Galb1-4GlcNAcb1-3Galb1-4GlcNAcb1-3Galb1-4GlcNAcb1-2Mana1-3)Manb1-4GlcNAcb1-4GlcNAcb-Sp25
564	Galb1-4GlcNAcb1-3Galb1-4GlcNAcb1-3Galb1-4GlcNAcb1-3Galb1-4GlcNAcb1-3Galb1-4GlcNAcb1-2Mana1-6(Galb1-4GlcNAcb1-3Galb1-4GlcNAcb1-3Galb1-4GlcNAcb1-3Galb1-4GlcNAcb1-3Galb1-4GlcNAcb1-2Mana1-3)Manb1-4GlcNAcb1-4GlcNAcb-Sp25
565	GlcNAb1-3Galb1-3GalNAc-Sp14
566	Galb1-3GlcNAcb1-6(Galb1-3)GalNAc-Sp14
567	Galb1-4GlcNAcb1-3Galb1-4GlcNAcb1-3Galb1-4GlcNAcb1-3Galb1-4GlcNAcb1-3Galb1-4GlcNAcb1-3Galb1-4GlcNAcb1-2Mana1-6(Galb1-4GlcNAcb1-3Galb1-4GlcNAcb1-3Galb1-4GlcNAcb1-3Galb1-4GlcNAcb1-3Galb1-4GlcNAcb1-3Galb1-4GlcNAcb1-2Mana1-3)Manb1-4GlcNAcb1-4GlcNAcb-Sp25
568	(3S)GlcAb1-3Galb1-4GlcNAcb1-3Galb1-4Glc-Sp0
569	(3S)GlcAb1-3Galb1-4GlcNAcb1-2Mana-Sp0
570	Galb1-3GlcNAcb1-3Galb1-4GlcNAcb1-3Galb1-4GlcNAcb1-6(Galb1-3GlcNAcb1-3Galb1-4GlcNAcb1-3Galb1-4GlcNAb1-2)Mana1-6(Galb1-3GlcNAcb1-3Galb1-4GlcNAcb1-3Galb1-4GlcNAcb1-2Mana1-3)Manb1-4GlcNAcb1-4(Fuca1-6)GlcNAcb-Sp24
571	Galb1-3GlcNAcb1-3Galb1-4GlcNAcb1-6(Galb1-3GlcNAcb1-3Galb1-4GlcNAb1-2)Mana1-6(Galb1-3GlcNAcb1-3Galb1-4GlcNAcb1-2Mana1-3)Manb1-4GlcNAcb1-4(Fuca1-6)GlcNAcb-Sp24
572	Neu5Aca2-8Neu5Aca2-3Galb1-3GalNAcb1-4(Neu5Aca2-3)Galb1-4Glc-Sp21
573	GlcNAcb1-3Galb1-4GlcNAcb1-2Mana1-6(GlcNAcb1-3Galb1-4GlcNAcb1-2Mana1-3)Manb1-4GlcNAcb1-4(Fuca1-6)GlcNAcb-Sp24
574	Galb1-4GlcNAcb1-3Galb1-4GlcNAcb1-2Mana1-6(Galb1-4GlcNAcb1-3Galb1-4GlcNAcb1-2Mana1-3)Manb1-4GlcNAcb1-4(Fuca1-6)GlcNAcb-Sp24
575	GlcNAcb1-3Galb1-4GlcNAcb1-3Galb1-4GlcNAcb1-2Mana1-6(GlcNAcb1-3Galb1-4GlcNAcb1-3Galb1-4GlcNAcb1-2Mana1-3)Manb1-4GlcNAcb1-4(Fuca1-6)GlcNAcb-Sp24
576	Galb1-4GlcNAcb1-3Galb1-4GlcNAcb1-3Galb1-4GlcNAcb1-2Mana1-6(Galb1-4GlcNAcb1-3Galb1-4GlcNAcb1-3Galb1-4GlcNAcb1-2Mana1-3)Manb1-4GlcNAcb1-4(Fuca1-6)GlcNAcb-Sp24
577	GlcNAcb1-3Galb1-4GlcNAcb1-3Galb1-4GlcNAcb1-3Galb1-4GlcNAcb1-2Mana1-6(GlcNAcb1-3Galb1-4GlcNAcb1-3Galb1-4GlcNAcb1-3Galb1-4GlcNAcb1-2Mana1-3)Manb1-4GlcNAcb1-4(Fuca1-6)GlcNAcb-Sp24
578	Galb1-4GlcNAcb1-3Galb1-4GlcNAcb1-3Galb1-4GlcNAcb1-3Galb1-4GlcNAcb1-2Mana1-6(Galb1-4GlcNAcb1-3Galb1-4GlcNAcb1-3Galb1-4GlcNAcb1-3Galb1-4GlcNAcb1-2Mana1-3)Manb1-4GlcNAcb1-4(Fuca1-6)GlcNAcb-Sp24

579	GlcNAcb1-3Galb1-4GlcNAcb1-3Galb1-4GlcNAcb1-3Galb1-4GlcNAcb1-3Galb1-4GlcNAcb1-2Mana1-6(GlcNAcb1-3Galb1-4GlcNAcb1-3Galb1-4GlcNAcb1-3Galb1-4GlcNAcb1-3Galb1-4GlcNAcb1-2Mana1-3)Manb1-4GlcNAcb1-4(Fuca1-6)GlcNAcb-Sp19
580	Galb1-4GlcNAcb1-3Galb1-4GlcNAcb1-3Galb1-4GlcNAcb1-3Galb1-4GlcNAcb1-3Galb1-4GlcNAcb1-2Mana1-6(Galb1-4GlcNAcb1-3Galb1-4GlcNAcb1-3Galb1-4GlcNAcb1-3Galb1-4GlcNAcb1-3Galb1-4GlcNAcb1-2Mana1-3)Manb1-4GlcNAcb1-4(Fuca1-6)GlcNAcb-Sp19
581	Galb1-4GlcNAcb1-3Galb1-4GlcNAcb1-6(Galb1-4GlcNAcb1-3Galb1-4GlcNAb1-2)Mana1-6(Galb1-4GlcNAcb1-3Galb1-4GlcNAcb1-2Mana1-3)Manb1-4GlcNAcb1-4(Fuca1-6)GlcNAcb-Sp24
582	GlcNAcb1-3Galb1-4GlcNAcb1-3Galb1-4GlcNAcb1-6(GlcNAcb1-3Galb1-4GlcNAcb1-3Galb1-4GlcNAb1-2)Mana1-6(GlcNAcb1-3Galb1-4GlcNAcb1-3Galb1-4GlcNAcb1-2Mana1-3)Manb1-4GlcNAcb1-4(Fuca1-6)GlcNAcb-Sp24
583	Galb1-4GlcNAcb1-3Galb1-4GlcNAcb1-3Galb1-4GlcNAcb1-6(Galb1-4GlcNAcb1-3Galb1-4GlcNAcb1-3Galb1-4GlcNAb1-2)Mana1-6(Galb1-4GlcNAcb1-3Galb1-4GlcNAcb1-3Galb1-4GlcNAcb1-2Mana1-3)Manb1-4GlcNAcb1-4(Fuca1-6)GlcNAcb-Sp24
584	GlcNAcb1-3Galb1-4GlcNAcb1-3Galb1-4GlcNAcb1-3Galb1-4GlcNAcb1-6(GlcNAcb1-3Galb1-4GlcNAcb1-3Galb1-4GlcNAcb1-3Galb1-4GlcNAb1-2)Mana1-6(GlcNAcb1-3Galb1-4GlcNAcb1-3Galb1-4GlcNAcb1-3Galb1-4GlcNAcb1-2Mana1-3)Manb1-4GlcNAcb1-4(Fuca1-6)GlcNAcb-Sp24
585	Galb1-4GlcNAcb1-3Galb1-4GlcNAcb1-3Galb1-4GlcNAcb1-3Galb1-4GlcNAcb1-6(Galb1-4GlcNAcb1-3Galb1-4GlcNAcb1-3Galb1-4GlcNAcb1-3Galb1-4GlcNAb1-2)Mana1-6(Galb1-4GlcNAcb1-3Galb1-4GlcNAcb1-3Galb1-4GlcNAcb1-3Galb1-4GlcNAcb1-2Mana1-3)Manb1-4GlcNAcb1-4(Fuca1-6)GlcNAcb-Sp24
586	GlcNAcb1-3Galb1-4GlcNAcb1-3Galb1-4GlcNAcb1-3Galb1-4GlcNAcb1-3Galb1-4GlcNAcb1-6(GlcNAcb1-3Galb1-4GlcNAcb1-3Galb1-4GlcNAcb1-3Galb1-4GlcNAcb1-3Galb1-4GlcNAb1-2)Mana1-6(GlcNAcb1-3Galb1-4GlcNAcb1-3Galb1-4GlcNAcb1-3Galb1-4GlcNAcb1-3Galb1-4GlcNAcb1-2Mana1-3)Manb1-4GlcNAcb1-4(Fuca1-6)GlcNAcb-Sp24
587	Galb1-4GlcNAcb1-3Galb1-4GlcNAcb1-3Galb1-4GlcNAcb1-3Galb1-4GlcNAcb1-3Galb1-4GlcNAcb1-6(Galb1-4GlcNAcb1-3Galb1-4GlcNAcb1-3Galb1-4GlcNAcb1-3Galb1-4GlcNAcb1-3Galb1-4GlcNAb1-2)Mana1-6(Galb1-4GlcNAcb1-3Galb1-4GlcNAcb1-3Galb1-4GlcNAcb1-3Galb1-4GlcNAcb1-3Galb1-4GlcNAcb1-2Mana1-3)Manb1-4GlcNAcb1-4(Fuca1-6)GlcNAcb-Sp24
588	Galb1-4GlcNAcb1-3Galb1-4GlcNAcb1-3GalNAca-Sp14
589	Galb1-4GlcNAcb1-3Galb1-4GlcNAcb1-6(Galb1-3)GalNAca-Sp14
590	Galb1-4GlcNAcb1-3Galb1-4GlcNAcb1-6(Galb1-4GlcNAcb1-3Galb1-4GlcNAcb1-3)GalNAca-Sp14
591	Neu5Aca2-3Galb1-4GlcNAcb1-3Galb1-4GlcNAcb1-3GalNAca-Sp14
592	GlcNAcb1-3Galb1-4GlcNAcb1-3GalNAca-Sp14
593	GlcNAcb1-3Galb1-4GlcNAcb1-6(Galb1-3)GalNAca-Sp14
594	GlcNAcb1-3Galb1-4GlcNAcb1-6(GlcNAcb1-3Galb1-4GlcNAcb1-3)GalNAca-Sp14
595	Neu5Aca2-3Galb1-4GlcNAcb1-3Galb1-4GlcNAcb1-6(Neu5Aca2-3Galb1-4GlcNAcb1-3Galb1-4GlcNAcb1-3)GalNAca-Sp14
596	Neu5Aca2-6Galb1-4GlcNAcb1-3Galb1-4GlcNAcb1-3GalNAca-Sp14
597	GlcNAcb1-3Galb1-4GlcNAcb1-3Galb1-4GlcNAcb1-3GalNAca-Sp14
598	Galb1-4GlcNAcb1-3Galb1-3GalNAca-Sp14
599	Neu5Aca2-3Galb1-4GlcNAcb1-3Galb1-4GlcNAcb1-6(Galb1-3)GalNAca-Sp14
600	Neu5Aca2-6Galb1-4GlcNAcb1-3Galb1-4GlcNAcb1-6(Galb1-3)GalNAca-Sp14
601	Neu5Aca2-6Galb1-4GlcNAcb1-6(Galb1-3)GalNAca-Sp14
602	Neu5Aca2-3Galb1-4GlcNAcb1-3Galb1-4GlcNAcb1-2Mana1-6(Neu5Aca2-3Galb1-4GlcNAcb1-3Galb1-4GlcNAcb1-2Mana1-3)Manb1-4GlcNAcb1-4GlcNAcb-Sp12
603	GlcNAcb1-6(Neu5Aca2-3Galb1-3)GalNAca-Sp14
604	Neu5Aca2-6Galb1-4GlcNAcb1-3Galb1-4GlcNAcb1-6(Neu5Aca2-6Galb1-4GlcNAcb1-3Galb1-4GlcNAcb1-3)GalNAca-Sp14
605	Neu5Aca2-6Galb1-4GlcNAcb1-3Galb1-4GlcNAcb1-3Galb1-4GlcNAcb1-2Mana1-6(Neu5Aca2-6Galb1-4GlcNAcb1-3Galb1-4GlcNAcb1-3Galb1-4GlcNAcb1-2Mana1-3)Manb1-4GlcNAcb1-4GlcNAcb-Sp12
606	Neu5Aca2-3Galb1-4GlcNAcb1-3Galb1-4GlcNAcb1-3Galb1-4GlcNAcb1-2Mana1-6(Neu5Aca2-3Galb1-4GlcNAcb1-3Galb1-4GlcNAcb1-3Galb1-4GlcNAcb1-2Mana1-3)Manb1-4GlcNAcb1-4GlcNAcb-Sp12
607	Neu5Aca2-6Galb1-4GlcNAcb1-3Galb1-4GlcNAcb1-2Mana1-6(Neu5Aca2-6Galb1-4GlcNAcb1-3Galb1-4GlcNAcb1-2Mana1-3)Manb1-4GlcNAcb1-4GlcNAcb-Sp12
608	GlcNAcb1-3Fuca-Sp21
609	Galb1-3GalNAcb1-4(Neu5Aca2-8Neu5Aca2-8Neu5Aca2-3)Galb1-4Glcb-Sp21
